# An alternative method to analyse the biomarker‐strategy design

**DOI:** 10.1002/sim.7940

**Published:** 2018-09-09

**Authors:** Cornelia Ursula Kunz, Thomas Jaki, Nigel Stallard

**Affiliations:** ^1^ Department of Mathematics and Statistics Lancaster University Lancaster UK; ^2^ Institute of Medical Biometry and Informatics University of Heidelberg Heidelberg Germany; ^3^ Biostatistics & Data Sciences Boehringer Ingelheim Pharma GmbH & Co. KG Ingelheim am Rhein Germany; ^4^ Warwick Medical School University of Warwick Coventry UK

**Keywords:** analysis strategy, biomarker, design, interaction, personalised medicine

## Abstract

Recent developments in genomics and proteomics enable the discovery of biomarkers that allow identification of subgroups of patients responding well to a treatment. One currently used clinical trial design incorporating a predictive biomarker is the so‐called biomarker strategy design (or marker‐based strategy design). Conventionally, the results from this design are analysed by comparing the mean of the biomarker‐led arm with the mean of the randomised arm. Several problems regarding the analysis of the data obtained from this design have been identified in the literature. In this paper, we show how these problems can be resolved if the sample sizes in the subgroups fulfil the specified orthogonality condition. We also propose a different analysis strategy that allows definition of test statistics for the biomarker‐by‐treatment interaction effect as well as for the classical treatment effect and the biomarker effect. We derive equations for the sample size calculation for the case of perfect and imperfect biomarker assays. We also show that the often used 1:1 randomisation does not necessarily lead to the smallest sample size. In addition, we provide point estimators and confidence intervals for the treatment effects in the subgroups. Application of our method is illustrated using a real data example.

## 1 INTRODUCTION

The focus of modern medicine has shifted from broad spectrum treatments to targeted therapeutics leading to new challenges for the design and analysis of clinical trials. Recent developments in genomics and proteomics enable the discovery of biomarkers that allow identification of subgroups of patients responding well to a treatment. Often, little is known about this subset of patients until well into large‐scale clinical trials.^1^

The impact of genomic variability is often assessed in a biomarker‐based design. The general question when using any of these designs is whether different treatments should be recommended for different subgroups of patients. Establishing clinical relevance of a biomarker test for guiding therapy decisions requires demonstrating that it can classify patients into distinct subgroups with different recommended managements.^2^ Several clinical trial designs using a biomarker to identify subgroups of patients likely to respond to a treatment have been proposed in the literature. The most commonly used designs are the enrichment design and the biomarker stratified design.(3, 4)

**Figure 1:**
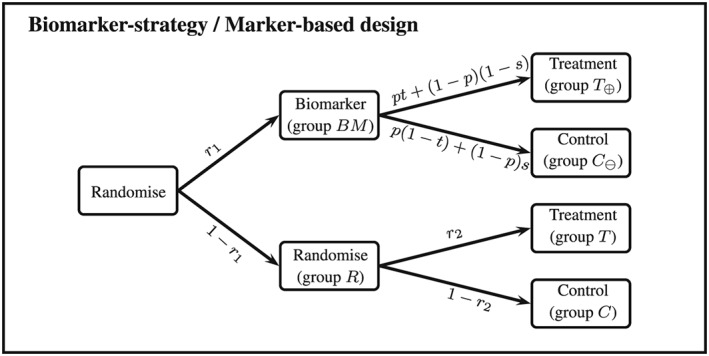
Biomarker‐strategy/marker‐based design

Another currently used design incorporating a predictive biomarker is the so‐called biomarker strategy design (or marker‐based strategy design).(2, 5, 6) Within this design, patients are randomised either to have their treatment based on the biomarker (ie, biomarker‐positive patients receive a new treatment while biomarker‐negative patients receive the standard treatment) or to be randomly assigned to treatment T or control group C (see Figure 1).

An alternative variant of the biomarker strategy design would not randomise patients in the randomised arm but treat everyone in this arm with the control treatment.(2, 3, 7) Although this design is a special case of the design described above (the probability to be randomised into the treatment arm is set to 0), this special case is more frequently used.^8^ The main criticism here is that a huge proportion of the patients receive the same treatment.^9^ Furthermore, given that all patients in the randomised arm receive the control treatment, this variant of the biomarker‐strategy design cannot establish whether the new treatment would be beneficial for biomarker‐negative patients as they all receive the control treatment.^3^

Conventionally, the results of a biomarker‐strategy design are analysed comparing the biomarker‐led arm (arm *B* *M*) to the randomised arm (arm *R*) (see Figure 1). The efficiency of this analysis has been investigated.(8, 10) However, all criticisms are focusing on special cases of the biomarker‐strategy design: Firstly, they all assume that randomisation to the biomarker‐led and the randomised arm is equal. Secondly, Hoering et al^8^ and Young et al^10^ both considered the special case of “no randomisation” in the randomised arm, which means that all patients in that arm receive the control treatment. Young et al also consider the case of equal randomisation within the randomised arm. While equal randomisation might be frequently used, it is not clear whether this minimises the sample size of the biomarker‐strategy design. Furthermore, Young et al investigate the efficiency of the design either if there is no treatment effect in the disease‐negative patients or if the treatment effect in the disease negative patients is in the same direction as the treatment effect for the positive patients but only half as large.

We hence extend the previous work in the following ways: (1) We investigate the efficiency of the design if the treatment effect in the disease‐negative patients is in the opposite direction of the treatment effect for the biomarker‐positive patients and (2) we investigate optimal randomisation rules for the design in order to minimise the sample size. Furthermore, we propose a different way to analyse the data obtained from the biomarker‐strategy design. The “traditional” analysis does not exploit all information that can be obtained from the data. Rücker^11^ proposed a two‐stage randomised clinical trial design, which in principle corresponds to the biomarker strategy design with the only difference that patients in the “non‐randomised arm” (ie, the biomarker‐led arm) choose the treatment they prefer. However, she suggests an analysis method that allows testing of the overall treatment effect, the self‐selection effect and the preference effect. Walter et al^12^ derive optimal randomisation rules for the abovementioned design by Rücker. Their focus lies only on optimising the first randomisation while they assume equal allocation to treatment and control in the randomised arm. Turner et al^13^ give a sample size equation for the design proposed by Rücker. However, like Walter et al, they also assume equal randomisation in the randomised arm. They also assume equal variances across all arms.

The aim of this paper is to adopt the analysis method proposed by Rücker and extend it to the biomarker strategy design by accounting for possible misclassifications using an imperfect assay. We provide equations to calculate the sample size as well as point estimators and confidence intervals for the treatment effects in the biomarker‐positive and biomarker‐negative subgroups.

## 2 MOTIVATING EXAMPLE

Brusselle et al^14^ report the results from the AZISAST trial, a multicentre randomised double‐blind placebo‐controlled trial. Patients with exacerbation‐prone severe asthma received low‐dose azithromycin or placebo as add‐on treatment to a combination therapy of inhaled corticosteroids and long‐acting *β*_2_ agonists for 6 months. The primary outcome was the rate of severe exacerbations and the lower respiratory tract infections (LRTI) requiring treatment with antibiotics during the 26‐week treatment phase, and one of the secondary endpoints was the forced expiratory volume in 1 second (*F* *E* *V*_1_). For the primary endpoint as well as the abovementioned secondary endpoint, no effects were found for the overall population. However, opposite effects were found for the primary endpoint for patients with either eosinophilic or non‐eosinophilic severe asthma. For the secondary outcome *F* *E* *V*_1_, they report a baseline value of 80.1 (standard deviation *S* *D* 21.9) for the azithromycin group and a baseline value of 84.8 (20.7) for the placebo group. They also report a mean difference (between baseline and 26 weeks) of −0.02 for the azithromycin group and a mean difference of −0.90 for the placebo group. They do not report the values for the two subgroups (eosinophilic or non‐eosinophilic severe asthma), so for our illustrations, we therefore assume the following values: $\mu_{T_{+}}=90$, $\mu_{T_{-}}=70$, $\mu_{C_{+}}=75$, and $\mu_{C_{-}}=95$. Patients with eosinophilic severe asthma are considered “positive.” Furthermore, we assume $\sigma_{T_{+}}=\sigma_{T_{-}}=\sigma_{C_{+}}=\sigma_{C_{-}}=20$. For a prevalence of *p* = 0.5 for eosinophilic asthma, the effect in the treatment group is $p\mu_{T_{+}}+(1-p)\mu_{T_{-}}=80$; for the control group, the effect is $p\mu_{C_{+}}+(1-p)\mu_{C_{-}}=85$; and the overall treatment effect is $p\mu_{T_{+}}+(1-p)\mu_{T_{-}}-(p\mu_{C_{+}}+(1-p)\mu_{C_{-}})=-5$, which roughly reflects the effects observed in the AZISAST trial.

## 3 NOTATION

Let *N* denote the total sample size (assumed to be fixed), *r*_1_ denote the fraction of patients randomised to the biomarker‐led arm, and let *n*_*BM*_ = *N* *r*_1_ denote the number of patients in the biomarker‐led arm. Furthermore, let *n*_*R*_ = *N*(1 − *r*_1_) denote the number of patients in the randomised arm. Let *r*_2_ denote the fraction of patients within the randomised arm that receive the experimental treatment and *n*_*T*_ = *N*(1 − *r*_1_)*r*_2_ denote the sample size for this arm. Furthermore, let *n*_*C*_ = *N*(1 − *r*_1_)(1 − *r*_2_) denote the sample size for the control arm of the randomised arm. We assume that a block randomisation is used so that the sample sizes *n*_*BM*_, *n*_*R*_, *n*_*T*_, and *n*_*C*_ are fixed.

Within the biomarker‐led arm, patients have their biomarker assessed. Due to cost, ethical, or administrative reasons, an imperfect assay is used to determine the true biomarker status. Let *p* denote the prevalence of the true biomarker status and let *t* and *s* denote the sensitivity and specificity of the biomarker assay used. Without loss of generality, we assume that patients with an observed positive biomarker status receive the experimental treatment while patients with an observed negative biomarker status receive the control treatment. Let $n_{T_{⊕}}$ ( $n_{C_{⊖}}$) denote the number of patients with an observed positive (negative) biomarker status. The sample sizes follow binomial distributions with $n_{T_{⊕}}∼Binomial(Nr_{1},p_{⊕})$ and $n_{C_{⊖}}∼Binomial(Nr_{1},p_{⊖})$ with *p*_⊕_ = *p* *t* + (1 − *p*)(1 − *s*) and *p*_⊖_ = *p*(1 − *t*) + (1 − *p*)*s* = 1 − *p*_⊕_.

The primary endpoint of interest is denoted with *Y* and follows a normal distribution with mean $\mu_{T_{+}}$ and variance $\sigma_{T_{+}}^{2}$ for truly biomarker‐positive patients receiving the experimental treatment, mean $\mu_{T_{-}}$ and variance $\sigma_{T_{-}}^{2}$ for truly biomarker‐negative patients receiving the experimental treatment, mean $\mu_{C_{+}}$ and variance $\sigma_{C_{+}}^{2}$ for truly biomarker‐positive patients receiving the control treatment, and mean $\mu_{C_{-}}$ and variance $\sigma_{C_{-}}^{2}$ for truly biomarker‐negative patients receiving the control treatment. Table 1 gives an overview of some of the notation used.

**Table 1:** Notation

		Biomarker (BM)‐led Group		
		Positive BM status	Negative BM status	Randomised group
Treatment	Index	*T*_⊕_		*T*
	True mean	$\theta_{T_{⊕}}=pt\mu_{T_{+}}+(1-p)(1-s)\mu_{T_{-}}$	…	$\mu_{T}=p\mu_{T_{+}}+(1-p)\mu_{T_{-}}$
	Sample size	$n_{T_{⊕}}$	…	*n*_*T*_
	Data	$\sum_{T_{⊕}}y=\sum_{i=1}^{n_{T_{⊕}}}y_{T_{⊕},i}$	…	$\sum_{T}y=\sum_{i=1}^{n_{T}}y_{T,i}$
Control	Index	…	*C*_⊖_	*C*
	True mean	…	$\theta_{C_{⊖}}=p(1-t)\mu_{C_{+}}+(1-p)s\mu_{C_{-}}$	$\mu_{C}=p\mu_{C_{+}}+(1-p)\mu_{C_{-}}$
	Sample size	…	$n_{C_{⊖}}$	*n*_*C*_
	Data	…	$\sum_{T_{⊖}}y=\sum_{i=1}^{n_{C_{⊖}}}y_{C_{⊖},i}$	$\sum_{C}y=\sum_{i=1}^{n_{C}}y_{C,i}$

## 4 TRADITIONAL ANALYSIS

The traditional analysis of the biomarker‐strategy design is to compare the mean of the biomarker‐led arm *μ*_*BM*_ with the mean of the randomised arm *μ*_*R*_.^15^ The null hypotheses states that $H_{0}^{TA}$: *μ*_*BM*_ = *μ*_*R*_ while the alternative hypothesis states that $H_{1}^{TA}$: *μ*_*BM*_ ≠ *μ*_*R*_.

Let *Z*_*BM*_ and *Z*_*R*_ be defined as $Z_{BM}=\frac{\sum_{T_{⊕}}y+\sum_{C_{⊖}}y}{Nr_{1}}$ and $Z_{R}=\frac{\sum_{T}y+\sum_{C}y}{N(1-r_{1})}$. A test statistic for the hypothesis above is then given by

(1) $$\frac{Z_{BM}-Z_{R}}{\sqrt{\hat{\mathrm{VAR}}\left[Z_{BM}-Z_{R}\right]}}.$$

The expected value and variance of the test statistic as well as an estimator for the variance can be found in Appendix A.

### 4.1 Criticism

Although the biomarker strategy design is sometimes regarded as the gold standard, there is also a lot of criticism regarding the trial design and the analysis of the data. The main problem with the traditional analysis is that it does not distinguish between a situation where there is only an overall treatment effect (but no treatment‐by‐biomarker interaction effect), a situation where there is only an interaction effect (but no treatment effect), and a situation where there is both a treatment and an interaction effect. This “inability” leads to the following problems:

• **Problem 1 “No interaction effect”**: As mentioned above, the traditional analysis cannot distinguish between the treatment and the interaction effect. If there is no interaction effect, we have $\mu_{T_{+}}-\mu_{C_{+}}=\mu_{T_{-}}-\mu_{C_{-}}=\mu_{T•}-\mu_{C•}$. In this case, the expected value of the test statistic for the traditional analysis is E[*μ*_*BM*_ − *μ*_*R*_] = (*t* *p* + (1 − *p*)(1 − *s*) − *r*_2_)(*μ*_*T*•_ − *μ*_*C*•_). So, as long as *t* *p* + (1 − *p*)(1 − *s*) − *r*_2_ ≠ 0 and *μ*_*T*•_ − *μ*_*C*•_ ≠ 0, we observe a difference between the mean of the biomarker‐led arm and the randomised arm even if there is no treatment‐by‐biomarker interaction effect.
• **Problem 2 “Biomarker assay not predictive”:** As Freidlin, McShane, and Korn^2^ noted before for a design with *r*_2_ = 0, it is still possible to observe a difference between the means even if the biomarker assay is not predictive, ie, *t* = *s* = 0.5. In general, if *t* = *s* = 0.5, the expected difference between the two arms is $\left(0.5-r_{2})(p(\mu_{T_{+}}-\mu_{C_{+}})+(1-p)(\mu_{T_{-}}-\mu_{C_{-}})\right)$, which is the same as (0.5 − *r*_2_) times the “overall treatment effect.” Hence, we would still observe a difference between the biomarker‐led arm and the control arm (randomised arm) as long as there is an “overall treatment effect” and 0.5 − *r*_2_ ≠ 0. If the observed difference is positive, we would recommend to use the assay to inform about the treatment a patient should receive although the assay is not predictive.
• **Problem 3 “Arbitrary randomisation”**: The expected value of the test statistic for the traditional analysis depends on the arbitrary randomisation ratio *r*_2_ since $E[\mu_{BM}-\mu_{R}]=tp(\mu_{T_{+}}-\mu_{C_{+}})+(1-s)(1-p)(\mu_{T_{-}}-\mu_{C_{-}})-r_{2}(p(\mu_{T_{+}}-\mu_{C_{+}})+(1-p)(\mu_{T_{-}}-\mu_{C_{-}}))$. It can be shown that if $r_{2}=(pt(\mu_{T_{+}}-\mu_{C_{+}})+(1-p)(1-s)(\mu_{T_{-}}-\mu_{C_{-}}))/(p(\mu_{T_{+}}-\mu_{C_{+}})+(1-p)(\mu_{T_{-}}-\mu_{C_{-}}))$, the differences between the two means are always zero (E[*μ*_*BM*_ − *μ*_*R*_] = 0) irrespective of the values of the other parameters.

### 4.2 Orthogonality condition

In the previous section, we have seen that the traditional analysis cannot distinguish between the treatment and the interaction effect. Under Problem 1, we show that the expected value in case there is no interaction effect is E[*μ*_*BM*_ − *μ*_*R*_] = (*t* *p* + (1 − *p*)(1 − *s*) − *r*_2_)(*μ*_*T*•_ − *μ*_*C*•_) = (*p*_⊕_ − *r*_2_)(*μ*_*T*•_ − *μ*_*C*•_). One way of solving this problem is to set *r*_2_ = *p*_⊕_. This ensures that the traditional analysis is now a test for the interaction effect only and that if there is no interaction effect, the expected value of the test statistic is indeed 0 (the proof can be found in Appendix A). Hence, setting $r_{2}=p_{T_{⊕}}$ is a requirement in order to be able to interpret the results of the traditional analysis.

We call this the “orthogonality condition” for the traditional analysis as it is similar to the “orthogonality condition” in a two‐way ANOVA, ie, sample sizes in the different cells have to be proportional (in our case, $n_{T_{⊕}}/n_{T}=n_{C_{⊖}}/n_{C}$) in order to distinguish between the different effects.^16^ This will also solve Problem 2 as setting *r*_2_ = *p*_⊕_ leads to $E[\mu_{BM}-\mu_{R}]=p(1-p)(s+t-1)(\mu_{T_{+}}-\mu_{C_{+}}-(\mu_{T_{-}}-\mu_{C_{-}}))$ so that if *t* = *s* = 0.5, E[*μ*_*BM*_ − *μ*_*R*_] = 0. Hence, we do not observe a difference between the means of the biomarker‐led arm and the randomised arm if the biomarker assay is of no use at all. We also solve Problem 3, as *r*_2_ is now fixed to *p*_⊕_.

In general, the expected difference between the mean of the biomarker‐led arm and the randomised arm can now only be zero if (a) *p* = 0 or *p* = 1, (b) *s* + *t* = 1, or (c) $\mu_{T_{+}}-\mu_{C_{+}}-(\mu_{T_{-}}-\mu_{C_{-}})=0$. Case (a) refers to a situation where there are no subgroups to distinguish as every patients is either truly positive or truly negative, case (b) refers to a situation where the biomarker assay is of no use to distinguish between truly positive and truly negative patients, and case (c) refers to a situation where there is no treatment‐by‐biomarker interaction effect. While setting *r*_2_ to *p*_⊕_ fixes the problems regarding the traditional analysis, in practice, the value of *p*_⊕_ is often unknown. Hence, a different method that is robust to the choice of *r*_2_ is preferable. Furthermore, there might be other reasons (ethical or financial) why a different value of *r*_2_ might be chosen.

## 5 ALTERNATIVE ANALYSIS METHOD

In order to overcome the abovementioned disadvantage of the traditional analysis method, we propose a different way to analyse the data obtained from a biomarker‐strategy design by defining three test statistics that clearly distinguish between the treatment effect, the (so‐called) biomarker effect, and the interaction effect.

### 5.1 Hypotheses

We define the following three effects: (1) the treatment effect, (2) the biomarker effect, and (3) the interaction effect with corresponding null hypotheses

(2) $$H_{0}^{T}:\mu_{T}-\mu_{C}=p\mu_{T_{+}}+(1-p)\mu_{T_{-}}-\left(p\mu_{C_{+}}+(1-p)\mu_{C_{-}}\right)=0,$$

(3) $$H_{0}^{B}:\frac{\mu_{T_{+}}+\mu_{C_{+}}}{2}-\frac{\mu_{T_{-}}+\mu_{C_{-}}}{2}=0,$$

(4) $$H_{0}^{I}:\frac{\mu_{T_{+}}-\mu_{C_{+}}}{2}-\frac{\mu_{T_{-}}-\mu_{C_{-}}}{2}=0.$$

### 5.2 Test statistics for the treatment, biomarker, and interaction effect

#### 5.2.1 Test statistic for the treatment effect

The two means *μ*_*T*_ and *μ*_*C*_ can be estimated directly from the design by $\frac{1}{n_{T}}\sum_{T}y=Z_{TR}$ and $\frac{1}{n_{C}}\sum_{C}y=Z_{CR}$, respectively. Hence, we can define a test statistic for the treatment effect as follows:

(5) $$T_{T}=\frac{Z_{TR}-Z_{CR}}{\sqrt{\hat{\mathrm{VAR}}\left[Z_{TR}-Z_{CR}\right]}}.$$

The expected value and variance of the test statistic as well as an estimator for the variance are given in Appendix B.

#### 5.2.2 Test statistics for the biomarker and the interaction effect

In the following, we derive test statistics for the biomarker and the interaction effect. Note that the null hypotheses for the biomarker and the interaction effect depend on the means $\mu_{T_{+}}$, $\mu_{C_{+}}$, $\mu_{T_{-}}$, and $\mu_{C_{-}}$, which cannot be estimated directly from the design. However, it is possible to rewrite the null hypotheses in such a way that only parameters that can be estimated directly from the data are involved. Let $\theta_{T_{⊕}}$ denote the true mean of the biomarker‐led treatment arm, and let $\theta_{C_{⊖}}$ denote the true mean of the biomarker‐led control arm (see Table 1). It can be shown that in the case that there is no biomarker (interaction) effect, the following expressions are true:

(6) $$\begin{array}{ccc} H_{0}^{B}:\;\;\;\;\;\;\;\;\;\;\;\;\;\;\;\;\;\frac{\mu_{T_{+}}+\mu_{C_{+}}}{2}-\frac{\mu_{T_{-}}+\mu_{C_{-}}}{2} & =0 & \\ \Leftrightarrow H_{0}^{B}:\theta_{T_{⊕}}-\mu_{T}(pt+(1-p)(1-s))-(\theta_{C_{⊖}}-\mu_{C}(p(1-t)+s(1-p))) & =0 \end{array}$$

(7) $$\begin{array}{ccc} H_{0}^{I}:\;\;\;\;\;\;\;\;\;\;\;\;\;\;\;\;\;\frac{\mu_{T_{+}}-\mu_{C_{+}}}{2}-\frac{\mu_{T_{-}}-\mu_{C_{-}}}{2} & =0 & \\ \Leftrightarrow H_{0}^{I}:\theta_{T_{⊕}}-\mu_{T}(pt+(1-p)(1-s))+(\theta_{C_{⊖}}-\mu_{C}(p(1-t)+s(1-p))) & =0. \end{array}$$

Now, let *Z*_*T*_ and *Z*_*C*_ be defined as follows:

(8) $$Z_{T}=\sum_{T_{⊕}}y-\frac{n_{T_{⊕}}}{n_{T}}\sum_{T}y$$

(9) $$Z_{C}=\sum_{C_{⊖}}y-\frac{n_{C_{⊖}}}{n_{C}}\sum_{C}y.$$

It can be shown that the left‐hand side of Equations 6 and 7 can be estimated by $\left(Z_{T}-Z_{C}\right)/\left(Nr_{1}\right)$ and $\left(Z_{T}+Z_{C}\right)/\left(Nr_{1}\right)$, respectively. Hence, a test statistic for the biomarker effect is given by

(10) $$T_{B}=\frac{Z_{T}-Z_{C}}{\sqrt{\hat{\mathrm{VAR}}\left[Z_{T}-Z_{C}\right]}}$$

and for the interaction effect by

(11) $$T_{I}=\frac{Z_{T}+Z_{C}}{\sqrt{\hat{\mathrm{VAR}}\left[Z_{T}+Z_{C}\right]}}.$$

The expected values and variances as well as estimators for the variances can be found in Appendix C.

### 5.3 Multiple testing

In cases where little is known about the new treatment under investigation and/or the biomarker used, it might be desirable to test more than one hypotheses within the same trial. For example, we might be interested in simultaneously testing the treatment and the interaction effect. Let COV_*T*,*B*_, COV_*T*,*I*_, and COV_*B*,*I*_ denote the covariances between the test statistics for the treatment and the biomarker effect (T,B), the treatment and the interaction effect (T,I), and the biomarker and the interaction effect (B,I). The expected values of the covariances are given by

(12) $${\text{COV}}_{T,B}=-\frac{\sqrt{N}r_{1}\left(\sigma_{T}^{2}(1-r_{2})(pt+(1-p)(1-s))+\sigma_{C}^{2}r_{2}(p(1-t)+(1-p)s)\right)}{\sqrt{(1-r_{1})r_{2}(1-r_{2})\left(\mathrm{VAR}\left[Z_{T}\right]+\mathrm{VAR}\left[Z_{C}\right]-2\text{COV}\left[Z_{T},Z_{C}\right]\right)\left(r_{2}\sigma_{C}^{2}+(1-r_{2})\sigma_{T}^{2}\right)}}$$

(13) $${\text{COV}}_{T,I}=-\frac{\sqrt{N}r_{1}\left(\sigma_{T}^{2}(1-r_{2})(pt+(1-p)(1-s))-\sigma_{C}^{2}r_{2}(p(1-t)+(1-p)s)\right)}{\sqrt{(1-r_{1})r_{2}(1-r_{2})\left(\mathrm{VAR}\left[Z_{T}\right]+\mathrm{VAR}\left[Z_{C}\right]+2\text{COV}\left[Z_{T},Z_{C}\right]\right)\left(r_{2}\sigma_{C}^{2}+(1-r_{2})\sigma_{T}^{2}\right)}}$$

(14) $${\text{COV}}_{B,I}=\frac{\mathrm{VAR}\left[Z_{T}\right]-\mathrm{VAR}\left[Z_{C}\right]}{\sqrt{\mathrm{VAR}\left[Z_{T}-Z_{C}\right]}\sqrt{\mathrm{VAR}\left[Z_{T}+Z_{C}\right]}}.$$

Let *T* denote the vector containing the three test statistics for the treatment, biomarker, and interaction effect with

$$T=\left(\begin{array}{c} T_{T}\\ T_{B}\\ T_{I} \end{array}\right)\overset{approx.}{∼}N\left(\mu ,\Sigma \right)$$

with

$$\mu =\left(\begin{array}{c} E\left[T_{T}\right]\\ E\left[T_{B}\right]\\ E\left[T_{I}\right] \end{array}\right),\Sigma =\left(\begin{array}{ccc} 1 & & \\ {\text{COV}}_{T,B} & 1 & \\ {\text{COV}}_{T,I} & {\text{COV}}_{B,I} & 1 \end{array}\right).$$

Functions like qmvnorm from the *R*‐Package mvtnorm or the Mata function ghk() from *Stata* can be used in order to find the adjusted critical values to control the overall type I error rate. However, in the following, we focus on testing the interaction effect only, so no adjustment is made.

### 5.4 Sample size calculation

In the following, we give formulae to approximately calculate the sample sizes to test the different hypotheses given above. To simplify the notation, let *σ*_*T*_, *σ*_*C*_, $\sigma_{T_{⊕}}$, $\sigma_{T_{⊖}}$, *A*, *B*, and *C* be defined as follows:

$$\begin{array}{ccc} \sigma_{T}^{2} & =p\left(\mu_{T_{+}}^{2}+\sigma_{T_{+}}^{2}\right)+(1-p)\left(\mu_{T_{-}}^{2}+\sigma_{T_{-}}^{2}\right)-\mu_{T}^{2} & \\ \sigma_{C}^{2} & =p\left(\mu_{C_{+}}^{2}+\sigma_{C_{+}}^{2}\right)+(1-p)\left(\mu_{C_{-}}^{2}+\sigma_{C_{-}}^{2}\right)-\mu_{C}^{2}\\ \sigma_{T_{⊕}}^{2} & =pt\left(\mu_{T_{+}}^{2}+\sigma_{T_{+}}^{2}\right)+(1-p)(1-s)\left(\mu_{T_{-}}^{2}+\sigma_{T_{-}}^{2}\right)-\theta_{T_{⊕}}^{2}\\ \sigma_{C_{⊖}}^{2} & =p(1-t)\left(\mu_{C_{+}}^{2}+\sigma_{C_{+}}^{2}\right)+(1-p)s\left(\mu_{C_{-}}^{2}+\sigma_{C_{-}}^{2}\right)-\theta_{C_{⊖}}^{2}\\ A & =\frac{\sigma_{T_{⊕}}^{2}+\sigma_{C⊖}^{2}+p_{⊕}p_{⊖}\left(\mu_{T}^{2}+\mu_{C}^{2}\right)-2\left(p_{⊖}\mu_{T}\theta_{T_{⊕}}+p_{⊕}\mu_{C}\theta_{C_{⊖}}\right)}{r_{1}}+\frac{(1-r_{2})p_{⊕}^{2}\sigma_{T}^{2}+r_{2}p_{⊖}^{2}\sigma_{C}^{2}}{\left(1-r_{1})r_{2}(1-r_{2}\right)}\\ B & =\frac{r_{1}r_{2}p_{⊕}p_{⊖}\sigma_{C}^{2}+r_{1}(1-r_{2})p_{⊕}p_{⊖}\sigma_{T}^{2}}{\left(1-r_{1})r_{2}(1-r_{2}\right)}\\ C & =-\theta_{T_{⊕}}\theta_{C_{⊖}}-p_{⊕}p_{⊖}\mu_{T}\mu_{C}+p_{⊖}\mu_{C}\theta_{T_{⊕}}+p_{⊕}\mu_{T}\theta_{C_{⊖}}. \end{array}$$

The following equations give the sample sizes required to detect a certain effect for the traditional analysis (*N*_*TR*_), the treatment effect (*N*_*T*_), the biomarker effect (*N*_*B*_), and the interaction effect (*N*_*I*_) based on a two two‐sided test with type I error *α* and power 1 − *β*:

(15) $$N_{TR}\approx {\left(\frac{z_{1-\alpha /2}+z_{1-\beta }}{\theta_{T_{⊕}}+\theta_{C_{⊖}}-\left(r_{2}\mu_{T}+(1-r_{2})\mu_{C}\right)}\right)}^{2}\left(\frac{\sigma_{T_{⊕}}^{2}+\sigma_{C_{⊖}}^{2}-2\theta_{T_{⊕}}\theta_{C_{⊖}}}{r_{1}}+\frac{r_{2}\sigma_{T}^{2}+(1-r_{2})\sigma_{C}^{2}}{1-r_{1}}\right)$$

(16) $$N_{T}\approx {\left(\frac{z_{1-\alpha /2}+z_{1-\beta }}{\mu_{T}-\mu_{C}}\right)}^{2}\frac{(1-r_{2})\sigma_{T}^{2}+r_{2}\sigma_{C}^{2}}{\left(1-r_{1})r_{2}(1-r_{2}\right)}$$

(17) $$\begin{array}{ccc} N_{B} & \approx {\left(\frac{z_{1-\alpha /2}+z_{1-\beta }}{\theta_{T_{⊕}}-\theta_{C_{⊖}}-p_{⊕}\mu_{T}+p_{⊖}\mu_{C}}\right)}^{2}\left(\frac{r_{1}A-2C}{2r_{1}}\right) & \\ & \;+\sqrt{{\left(\frac{z_{1-\alpha /2}+z_{1-\beta }}{\theta_{T_{⊕}}-\theta_{C_{⊖}}-p_{⊕}\mu_{T}+p_{⊖}\mu_{C}}\right)}^{4}{\left(\frac{r_{1}A-2C}{2r_{1}}\right)}^{2}+{\left(\frac{z_{1-\alpha /2}+z_{1-\beta }}{\theta_{T_{⊕}}-\theta_{C_{⊖}}-p_{⊕}\mu_{T}+p_{⊖}\mu_{C}}\right)}^{2}\frac{B}{r_{1}^{2}}} \end{array}$$

(18) $$\begin{array}{ccc} N_{I} & \approx {\left(\frac{z_{1-\alpha /2}+z_{1-\beta }}{\theta_{T_{⊕}}+\theta_{C_{⊖}}-p_{⊕}\mu_{T}-p_{⊖}\mu_{C}}\right)}^{2}\left(\frac{r_{1}A+2C}{2r_{1}}\right) & \\ & \;+\sqrt{{\left(\frac{z_{1-\alpha /2}+z_{1-\beta }}{\theta_{T_{⊕}}+\theta_{C_{⊖}}-p_{⊕}\mu_{T}-p_{⊖}\mu_{C}}\right)}^{4}{\left(\frac{r_{1}A+2C}{2r_{1}}\right)}^{2}+{\left(\frac{z_{1-\alpha /2}+z_{1-\beta }}{\theta_{T_{⊕}}+\theta_{C_{⊖}}-p_{⊕}\mu_{T}-p_{⊖}\mu_{C}}\right)}^{2}\frac{B}{r_{1}^{2}}} \end{array}$$

It can be seen that all sample sizes depend on the randomisation ratios *r*_1_ and *r*_2_. Optimal solutions can be found using a grid search.

### 5.5 Point estimators and confidence intervals

The test statistic for the interaction effect does not provide an answer to whether the interaction is quantitative (i.e., the treatment effect for biomarker‐positive and biomarker‐negative patients points in the same direction but has a different magnitude) or qualitative (i.e., the treatment effects for the two subgroups point in different directions). Hence, we derive point estimators and confidence intervals for the treatment effects $\mu_{T_{+}}-\mu_{C_{+}}$ and $\mu_{T_{-}}-\mu_{C_{-}}$ for the biomarker‐positive and biomarker‐negative patients, respectively. In the following, we assume that the true values of the prevalence *p*, the sensitivity *t*, and the specificity *s* are known (or can be estimated from a different trial). The treatment effects for biomarker‐positive and biomarker‐negative patients can be estimated by

(19) $${\hat{\mu }}_{T_{+}}-{\hat{\mu }}_{C_{+}}=\frac{\frac{1}{Nr_{1}}\sum_{T_{⊕}}y+\frac{1}{Nr_{1}}\sum_{C_{⊖}}y-(1-s)\frac{1}{n_{T}}\sum_{T}y-s\frac{1}{n_{C}}\sum_{C}y}{p(t+s-1)},$$

(20) $${\hat{\mu }}_{T_{-}}-{\hat{\mu }}_{C_{-}}=-\frac{\frac{1}{Nr_{1}}\sum_{T_{⊕}}y+\frac{1}{Nr_{1}}\sum_{C_{⊖}}y-t\frac{1}{n_{T}}\sum_{T}y-(1-t)\frac{1}{n_{C}}\sum_{C}y}{\left(1-p)(t+s-1\right)}.$$

Assuming that the estimators approximately follow a normal distribution, (1 − *α*)% confidence intervals are given by

(21) $$\left[{\hat{\mu }}_{T_{+}}-{\hat{\mu }}_{C_{+}}-z_{1-\alpha /2}\sqrt{\hat{\mathrm{VAR}}\left[{\hat{\mu }}_{T_{+}}-{\hat{\mu }}_{C_{+}}\right]},{\hat{\mu }}_{T_{+}}-{\hat{\mu }}_{C_{+}}+z_{1-\alpha /2}\sqrt{\hat{\mathrm{VAR}}\left[{\hat{\mu }}_{T_{+}}-{\hat{\mu }}_{C_{+}}\right]}\right]$$

(22) $$\left[{\hat{\mu }}_{T_{-}}-{\hat{\mu }}_{C_{-}}-z_{1-\alpha /2}\sqrt{\hat{\mathrm{VAR}}\left[{\hat{\mu }}_{T_{-}}-{\hat{\mu }}_{C_{-}}\right]},{\hat{\mu }}_{T_{-}}-{\hat{\mu }}_{C_{-}}+z_{1-\alpha /2}\sqrt{\hat{\mathrm{VAR}}\left[{\hat{\mu }}_{T_{-}}-{\hat{\mu }}_{C_{-}}\right]}\right]$$

with

(23) $$\begin{array}{ccc} \hat{\mathrm{VAR}}\left[{\hat{\mu }}_{T_{+}}-{\hat{\mu }}_{C_{+}}\right] & =\frac{1}{{\left(p(t+s-1)\right)}^{2}}\left(\frac{Nr_{1}\sum_{T_{⊕}}y^{2}-{\left(\sum_{T_{⊕}}y\right)}^{2}}{{\left(Nr_{1}\right)}^{2}(Nr_{1}-1)}+\frac{Nr_{1}\sum_{C_{⊖}}y^{2}-{\left(\sum_{C_{⊖}}y\right)}^{2}}{{\left(Nr_{1}\right)}^{2}(Nr_{1}-1)}\right. & \\ & \;\left.+\frac{{\left(1-s\right)}^{2}s_{T}^{2}}{n_{T}}+\frac{s^{2}s_{C}^{2}}{n_{C}}-\frac{2}{{\left(Nr_{1}\right)}^{2}(Nr_{1}-1)}\sum_{T_{⊕}}y\sum_{C_{⊖}}y\right) \end{array}$$

(24) $$\begin{array}{ccc} \hat{\mathrm{VAR}}\left[{\hat{\mu }}_{T_{-}}-{\hat{\mu }}_{C_{-}}\right] & =\frac{1}{{\left(\left(1-p)(t+s-1\right)\right)}^{2}}\left(\frac{Nr_{1}\sum_{T_{⊕}}y^{2}-{\left(\sum_{T_{⊕}}y\right)}^{2}}{{\left(Nr_{1}\right)}^{2}(Nr_{1}-1)}+\frac{Nr_{1}\sum_{C_{⊖}}y^{2}-{\left(\sum_{C_{⊖}}y\right)}^{2}}{{\left(Nr_{1}\right)}^{2}(Nr_{1}-1)}\right. & \\ & \;\left.+\frac{t^{2}s_{T}^{2}}{n_{T}}+\frac{{\left(1-t\right)}^{2}s_{C}^{2}}{n_{C}}-\frac{2}{{\left(Nr_{1}\right)}^{2}(Nr_{1}-1)}\sum_{T_{⊕}}y\sum_{C_{⊖}}y\right). \end{array}$$

The derivations can be found in the Supporting Information where we also provide the covariance between the two estimators allowing for the construction of joint confidence regions.

## 6 RESULTS

In order to be able to compare the traditional and the alternative analysis method, we set *r*_2_ = *p*_⊕_. As shown in Section 4.2, this will ensure that the traditional analysis method provides a test statistic for the interaction effect only. On the basis of the results for the AZISAST trial (see Section 2), we calculated the sample sizes to test the interaction effect using the traditional and the alternative analysis method for different values of the prevalence *p* (from 0 to 1 in steps of 0.01) and the sensitivity *t* and specificity *s* (from 0.5 to 1 in steps of 0.1) for a two‐sided type I error of *α* = 0.05 and a power of 80*%*. In the following, we focus on testing the interaction effect only (instead of testing the treatment, the biomarker, and the interaction effect simultaneously), so no adjustment for the significance level is made. For the traditional analysis, *r*_2_ is set to *p*_⊕_ (as explained in Section 4.2). In order to find the optimal value for *r*_1_, we calculated the sample sizes for values of *r*_1_ from 0 to 1 in steps of 0.01. The randomisation ratio *r*_1_ is then chosen so that the resulting sample size is minimised. Obviously, choosing smaller increments will yield a more accurate value for *r*_1_. For the alternative analysis method, we used a grid search over *r*_1_ and *r*_2_ (from 0 to 1 in steps of 0.01). For each combination of *r*_1_ and *r*_2_, we calculated the resulting sample sizes and chose the combination that minimises the sample size for given values of *p*, *t*, and *s*. In order to verify the obtained sample sizes, we simulated 10 000 data sets and estimated the resulting type I error and power for different scenarios (see Appendix D).

### 6.1 Results for the traditional analysis to test the interaction effect

**Figure 2:**
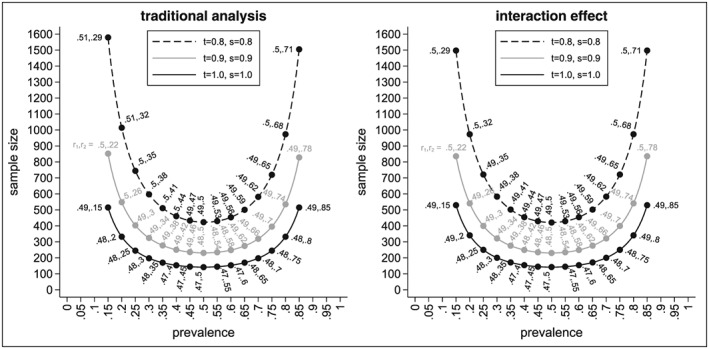
Minimal sample sizes to test the interaction effect using the traditional and alternative analysis method depending on the prevalence p, the sensitivity t, the specificity s, and the randomisation ratios r_1_ and r_2_

The left‐hand side of Figure 2 shows the minimal sample sizes needed for the traditional analysis depending on the prevalence *p*, the sensitivity *t*, and the specificity *s* for the AZISAST trial. The black solid line shows the sample sizes for a perfect biomarker assay, the grey solid line for a biomarker assay with *t* = *s* = 0.9, and the black dashed line for a biomarker assay with *t* = *s* = 0.8. The labels show the values for the randomisation ratios *r*_1_ and *r*_2_ with the value of *r*_1_ being the optimal value for *r*_2_ = *p*_⊕_.

As expected, we see that the sample size for the perfect assay is smaller than the sample sizes for an imperfect assay with the sample size getting larger the smaller the values for *t* and *s*. We also see that the sample size increases with smaller and larger values for the prevalence *p*. The sample size for the traditional analysis is smallest for a perfect assay, a prevalence of *p* = 0.5, and randomisation ratios of *r*_1_ = 0.47 and *r*_2_ = 0.5. In this case, only 142 patients would be needed. If the prevalence is 0.15, the sample size increases to 516 for the perfect biomarker assay.

For an imperfect assay with *t* = *s* = 0.9, the sample size is 231 for a prevalence of 0.5, and hence, in comparison to a perfect assay, we already need nearly 90 patients more. For a prevalence of 0.15, the sample size increases to 853, and therefore, nearly 340 more patients are needed in comparison with the perfect assay. The sample sizes are even larger when the sensitivity and specificity are 0.8. In this case, 423 patients are needed if *p* = 0.5 and 1580 if *p* = 0.15. Compared with the case of a perfect assay, the sample sizes approximately triple.

In general, it should be noted that the optimal value for *r*_1_ is not necessarily 0.5 but varies between 0.47 and 0.51. However, for a prevalence between 0.05 and 0.95, the difference in sample sizes for the optimal value of *r*_1_ and *r*_1_ = 0.5 is less than one patient. Therefore, in most cases, *r*_1_ can be set to 0.5 without a noticeable change in the sample size.

### 6.2 Results for the alternative method to test the interaction effect

In the following, we show the results for the sample sizes for testing the interaction effect based on our proposed analysis method. The right‐hand side of Figure 2 shows the resulting sample sizes for the interaction effect. As before, the black solid line shows the sample sizes for the perfect biomarker assay, the grey solid line for an assay with *t* = *s* = 0.9, and the black dashed line for an assay with *t* = *s* = 0.8. Labels show the optimal values for *r*_1_ and *r*_2_ so that the sample size is minimised for given values of *p*, *t*, and *s*.

Again, we see that the sample sizes are smaller for prevalences around 0.5 and increase towards smaller and larger values. For example, for a perfect assay and a prevalence of 0.5, the minimal sample size for the interaction effect is 142. It increases to 250 if the prevalence is 0.25 and to 530 if the prevalence is only 0.15. We also see that (as expected) larger sample sizes are needed if the assay is not perfect. For example, for an imperfect assay with *t* = *s* = 0.9, the minimal sample sizes are 231 (for *p* = 0.5), 400 (for *p* = 0.25), and 836 (for *p* = 0.15). For an imperfect assay with *t* = *s* = 0.8, the following sample sizes result 422 (for *p* = 0.5), 722 (for *p* = 0.25), and 1498 (for *p* = 0.15).

As for the traditional analysis, we note that the optimal value for the randomisation ratio *r*_2_ is *r*_2_ = *p*_⊕_. However, while this is a requirement for the traditional analysis in order for the results to be interpretable (see Section 4.2), for the interaction effect setting *r*_2_ to *p*_⊕_ minimises the sample size but is not necessary in order to interpret the results of the test statistic.

**Figure 3:**
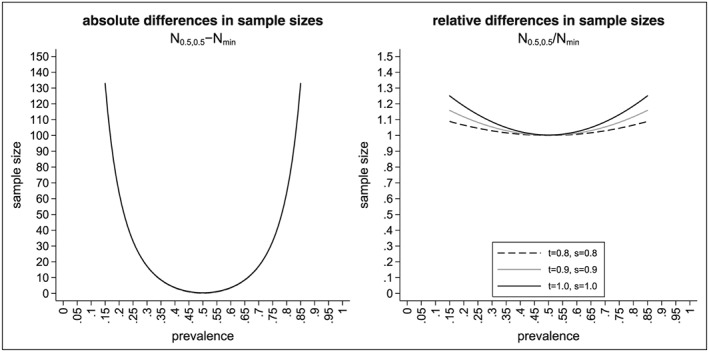
Difference in sample sizes to test the interaction effect using the alternative analysis method with r_1_ = r_2_ = 0.5 or the optimal randomisation ratios depending on the prevalence p, the sensitivity t, the specificity s, and the randomisation ratios r_1_ and r_2_

**Figure 4:**
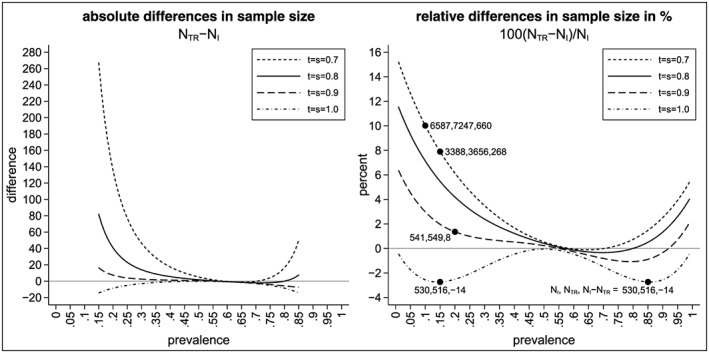
Differences in sample sizes to test the interaction effect using the traditional and alternative analysis method depending on the prevalence p, the sensitivity t, and the specificity s

We also see that the optimal value for *r*_1_ is not necessarily 0.5 but ranges between 0.47 and 0.5. Again, the differences between the minimal sample size and the one obtained for *r*_1_ = 0.5 is less than one patient as long as the second randomisation ratio *r*_2_ is chosen so that the sample size is minimised given *r*_1_. The situation changes noticeably if both randomisation rules are set to 0.5, which is frequently done. Let *N*_0.5,0.5_ denote the sample size for the interaction effect for *r*_1_ = *r*_2_ = 0.5 and let *N*_*min*_ denote the minimal sample size. Figure 3 shows the absolute difference in the sample sizes (*N*_0.5,0.5_ − *N*_*min*_, left‐hand side) and the relative differences (*N*_0.5,0.5_/*N*_*min*_, right‐hand side). As we can see, the absolute differences vary between 0 and approximately 130 (for a prevalence between 0.15 and 0.85). The absolute difference is even larger for smaller (and higher) values of the prevalence. While we see that the absolute difference of the sample sizes does not depend on the sensitivity and specificity of the biomarker, we see that the relative difference does (see right‐hand side of Figure 3). The highest relative differences occur for the perfect biomarker assay (as this has the smallest minimal sample size). Figure 4 shows the absolute differences in sample size for the traditional analysis and the alternative analysis for the interaction effect (left‐hand side) as well as the relative differences in sample size in percent (right‐hand side) . As we can see, the sample size for the traditional analysis is often higher than the sample size for the interaction effect (except for a perfect biomarker assay where the sample size for the traditional design is always lower than the sample size for the interaction effect). The absolute difference in the sample size might be substantial: for example, for *t* = *s* = 0.7 and *p* = 0.15, the absolute difference in sample size is 268. The relative increase in the sample size is about 8% (from *N*_*I*_ = 3388 to *N*_*TR*_ = 3656). We can also see that in cases where the sample size for the traditional analysis is smaller than the sample size for the interaction effect, the increase in sample size is less than 3%. For example, for *t* = *s* = 1 and *p* = 0.15, the sample size increases from *N*_*TR*_ = 516 to *N*_*I*_ = 530.

## 7 CONCLUSION

This paper investigates the performance of the traditional analysis of the biomarker‐strategy design. We derive optimal randomisation rules in order to minimise the sample size for the traditional analysis. We also propose a different analysis method for the data obtained from such a design and explore the properties of this method.

The traditional analysis has been criticised many times.(2, 9, 17, 18) The main problem is that the traditional analysis does not distinguish between the treatment and the interaction effect. Hence, it is possible to observe no difference between the mean of the biomarker‐led arm and the randomised arm even if there is an interaction effect or to observe a difference between the means even if there is no interaction effect. We show that if the sample sizes in the four subgroups of the biomarker‐strategy design fulfil the “orthogonality condition” (ie, are proportional to each other), the abovementioned problems can be solved. However, while the “orthogonality condition” ensures that the traditional analysis provides a test statistic for the interaction effect only, the value of *p*_⊕_ is often not known as it depends on the true values for the sensitivity, the specificity, and the prevalence. These values are often unknown at the planning stage of a trial.

To overcome the disadvantages of the traditional analysis, we propose a different analysis method based on the work of Rücker.^11^ We define three different effects than can be measured within the biomarker‐strategy design: the treatment effect, the biomarker effect, and the interaction effect. This ensures that our analysis can clearly distinguish between the treatment effect and the interaction effect, regardless of the randomisation rules used. We derive test statistics and show how the sample sizes for the three effects can be calculated. Optimal randomisation rules are derived in order to minimise the sample sizes. We also provide point estimators and confidence intervals for the treatment effects in the two subgroups.

Furthermore, we show that if the “orthogonality condition” is fulfilled, the sample size for the interaction effect (based on the alternative analysis method) is often smaller than the sample size for the traditional analysis. In cases where the sample size for the alternative analysis method is larger than the sample size for the traditional analysis, the difference is less than 3% of the sample size and therefore often negligible. In general, we therefore recommend to use the alternative analysis method instead of the traditional analysis method.

It should be noted that the biomarker‐strategy design is less efficient than the biomarker‐stratified design. Shih and Lin^19^ recently published an evaluation of the relative efficiency comparing the variances of the two designs with each other. They conclude that the biomarker‐strategy design is less efficient than the biomarker‐stratified design, which comes as no surprise given that the biomarker‐stratified design contains more information. While the qualitative conclusion of Shih and Lin is correct (ie, the biomarker‐stratified design is more efficient than the biomarker‐strategy design), their quantitative results are based on setting *r*_2_ equal to 0.5. As we have shown, this choice will not lead to the most efficient design (see Section 6.2). Hence, it remains to be seen how much more efficient the biomarker‐stratified design is as compared to the biomarker‐strategy design if optimal randomisation rules are used.

## CONFLICT OF INTEREST

The authors declare no potential conflict of interests.

## Supporting information



Supporting info item

## APPENDIX A TRADITIONAL ANALYSIS

### A.1

#### A.1.1

The expected value and variance of the test statistic for the traditional analysis are given by

(A1) $$\begin{array}{ccc} E\left[Z_{BM}-Z_{R}\right] & =(t-r_{2})p\left(\mu_{T_{+}}-\mu_{C_{+}}\right)+(1-s-r_{2})(1-p)\left(\mu_{T_{-}}-\mu_{C_{-}}\right) & \\ & =tp\left(\mu_{T_{+}}-\mu_{C_{+}}\right)+(1-s)(1-p)\left(\mu_{T_{-}}-\mu_{C_{-}}\right)-r_{2}\left(p\left(\mu_{T_{+}}-\mu_{C_{+}}\right)+(1-p)\left(\mu_{T_{-}}-\mu_{C_{-}}\right)\right) \end{array}$$

(A2) $$\begin{array}{ccc} \mathrm{VAR}\left[Z_{BM}-Z_{R}\right] & =\frac{pt\left(\mu_{T_{+}}^{2}+\sigma_{T_{+}}^{2}\right)+(1-p)(1-s)\left(\mu_{T_{-}}^{2}+\sigma_{T_{-}}^{2}\right)-{\left(pt\mu_{T_{+}}+(1-p)(1-s)\mu_{T_{-}}\right)}^{2}}{Nr_{1}} & \\ & \;+\frac{p(1-t)\left(\mu_{C_{+}}^{2}+\sigma_{C_{+}}^{2}\right)+(1-p)s\left(\mu_{C_{-}}^{2}+\sigma_{C_{-}}^{2}\right)-{\left(p(1-t)\mu_{C_{+}}+(1-p)s\mu_{C_{-}}\right)}^{2}}{Nr_{1}}\\ & \;-\frac{2\left(tp\mu_{T_{+}}+(1-p)(1-s)\mu_{T_{-}}\right)\left((1-t)p\mu_{C_{+}}+s(1-p)\mu_{C_{-}}\right)}{Nr_{1}}\\ & \;+\frac{\left[p\left(\mu_{T_{+}}^{2}+\sigma_{T_{+}}^{2}-\left(\mu_{C_{+}}^{2}+\sigma_{C_{+}}^{2}\right)\right)+(1-p)\left(\mu_{T_{-}}^{2}+\sigma_{T_{-}}^{2}-\left(\mu_{C_{-}}^{2}+\sigma_{C_{-}}^{2}\right)\right)\right]r_{2}}{N(1-r_{1})}\\ & \;-\frac{\left[{\left(p\mu_{T_{+}}+(1-p)\mu_{T_{-}}\right)}^{2}-{\left(p\mu_{C_{+}}+(1-p)\mu_{C_{-}}\right)}^{2}\right]r_{2}}{N(1-r_{1})}\\ & \;+\frac{p\left(\mu_{C_{+}}^{2}+\sigma_{C_{+}}^{2}\right)+(1-p)\left(\mu_{C_{-}}^{2}+\sigma_{C_{-}}^{2}\right)-{\left(p\mu_{C_{+}}+(1-p)\mu_{C_{-}}\right)}^{2}}{N(1-r_{1})}. \end{array}$$

The variance can be estimated by

(A3) $$\begin{array}{ccc} \hat{\mathrm{VAR}}\left[Z_{BM}-Z_{R}\right] & =\frac{\sum_{T_{⊕}}y^{2}-\frac{{\left(\sum_{T_{⊕}}y\right)}^{2}-\sum_{T_{⊕}}y^{2}}{Nr_{1}-1}+\sum_{C_{⊖}}y^{2}-\frac{{\left(\sum_{C_{⊖}}y\right)}^{2}-\sum_{C_{⊖}}y^{2}}{Nr_{1}-1}-2\frac{\sum_{T_{⊕}}y\sum_{C_{⊖}}y}{Nr_{1}}}{{\left(Nr_{1}\right)}^{2}} & \\ & \;+\frac{N(1-r_{1})r_{2}\sum_{T}y^{2}-{\left(\sum_{T}y\right)}^{2}}{{\left(N(1-r_{1})\right)}^{2}}+\frac{N(1-r_{1})(1-r_{2})\sum_{C}y^{2}-{\left(\sum_{C}y\right)}^{2}}{{\left(N(1-r_{1})\right)}^{2}}. \end{array}$$

In the following, we show that by setting *r*_2_ = *p*_⊕_, the traditional analysis method provides a test statistic for the interaction effect. According to Equation A1, the expected value is given by

(A4) $$\begin{array}{ccc} E\left[Z_{BM}-Z_{R}\right] & =tp\left(\mu_{T_{+}}-\mu_{C_{+}}\right)+(1-s)(1-p)\left(\mu_{T_{-}}-\mu_{C_{-}}\right)-r_{2}\left(p\left(\mu_{T_{+}}-\mu_{C_{+}}\right)+(1-p)\left(\mu_{T_{-}}-\mu_{C_{-}}\right)\right) & \\ & =\left(\mu_{T_{+}}-\mu_{C_{+}}\right)p\left(t-r_{2}\right)-\left(\mu_{T_{-}}-\mu_{C_{-}}\right)(1-p)\left(r_{2}-1+s\right)\\ & \overset{r_{2}=p_{⊕}}{=}\left(\mu_{T_{+}}-\mu_{C_{+}}\right)p\left(t-pt-(1-p)(1-s)\right)-\left(\mu_{T_{-}}-\mu_{C_{-}}\right)(1-p)\left(pt+(1-p)(1-s)-1+s\right)\\ & =2p(1-p)(t+s-1)\left(\frac{\mu_{T_{+}}-\mu_{C_{+}}}{2}-\frac{\mu_{T_{-}}-\mu_{C_{-}}}{2}\right). \end{array}$$

## APPENDIX B TREATMENT EFFECT

### B.1

#### B.1.1

The expected value and true variance of the test statistic for the treatment effect are given by

(B5) $$E\left[Z_{TR}-Z_{CR}\right]=p\left(\mu_{T_{+}}-\mu_{C_{+}}\right)+(1-p)\left(\mu_{T_{-}}-\mu_{C_{-}}\right)$$

(B6) $$\begin{array}{ccc} \mathrm{VAR}\left[Z_{TR}-Z_{CR}\right] & =\frac{p\left(\mu_{T_{+}}^{2}+\sigma_{T_{+}}^{2}\right)+(1-p)\left(\mu_{T_{-}}^{2}+\sigma_{T_{-}}^{2}\right)-{\left(p\mu_{T_{+}}+(1-p)\mu_{T_{-}}\right)}^{2}}{N(1-r_{1})r_{2}} & \\ & \;+\frac{p\left(\mu_{C_{+}}^{2}+\sigma_{C_{+}}^{2}\right)+(1-p)\left(\mu_{C_{-}}^{2}+\sigma_{C_{-}}^{2}\right)-{\left(p\mu_{C_{+}}+(1-p)\mu_{C_{-}}\right)}^{2}}{N(1-r_{1})(1-r_{2})}. \end{array}$$

The variance can be estimated by

(B7) $$\begin{array}{ccc} \hat{\mathrm{VAR}}\left[Z_{TR}-Z_{CR}\right] & = & =\frac{\sum_{T}y^{2}-\frac{1}{N(1-r_{1})r_{2}}{\left(\sum_{T}y\right)}^{2}}{\left(N(1-r_{1})r_{2})(N(1-r_{1})r_{2}-1\right)}+\frac{\sum_{C}y^{2}-\frac{1}{N(1-r_{1})(1-r_{2})}{\left(\sum_{C}y\right)}^{2}}{\left(N(1-r1)(1-r2))(N(1-r1)(1-r2)-1\right)}. \end{array}$$

## APPENDIX C BIOMARKER AND INTERACTION EFFECT

### C.1

#### C.1.1

The expected values are given by

(C8) $$E\left[Z_{T}\right]=Nr_{1}\theta_{T_{⊕}}-Nr_{1}\mu_{T}(pt+(1-p)(1-s))$$

(C9) $$E\left[Z_{C}\right]=Nr_{1}\theta_{C_{⊖}}-Nr_{1}\mu_{C}(p(1-t)+s(1-p)).$$

The true variances are given by

(C10) $$\begin{array}{ccc} \mathrm{VAR}\left[Z_{T}\right] & =Nr_{1}\left(pt\left(\mu_{T_{+}}^{2}+\sigma_{T_{+}}^{2}\right)+(1-p)(1-s)\left(\mu_{T_{-}}^{2}+\sigma_{T_{-}}^{2}\right)-{\left(pt\mu_{T_{+}}+(1-p)(1-s)\mu_{T_{-}}\right)}^{2}\right) & \\ & \;+Nr_{1}p_{+}(1-p_{+})\mu_{T}^{2}+({\left(Nr_{1}p_{+}\right)}^{2}+Nr_{1}p_{+}(1-p_{+}))\frac{\sigma_{T}^{2}}{N(1-r_{1})r_{2}}\\ & \;-2\left(Nr_{1}\mu_{T}(tp\mu_{T_{+}}+(1-p)(1-s)\mu_{T_{-}})(1-p_{+})\right) \end{array}$$

(C11) $$\begin{array}{ccc} \mathrm{VAR}\left[Z_{C}\right] & =Nr_{1}\left(p(1-t)\left(\mu_{C_{+}}^{2}+\sigma_{C_{+}}^{2}\right)+(1-p)s\left(\mu_{C_{-}}^{2}+\sigma_{C_{-}}^{2}\right)-{\left(p(1-t)\mu_{C_{+}}+(1-p)s\mu_{C_{-}}\right)}^{2}\right) & \\ & \;+Nr_{1}p_{-}(1-p_{-})\mu_{C}^{2}+\left({\left(Nr_{1}p_{-}\right)}^{2}+Nr_{1}p_{-}(1-p_{-})\right)\frac{\sigma_{C}^{2}}{N(1-r_{1})(1-r_{2})}\\ & \;-2\left(Nr_{1}\mu_{C}((1-t)p\mu_{C_{+}}+(1-p)s\mu_{C_{-}})(1-p_{-})\right) \end{array}$$

and the true covariance by

(C12) $$\begin{array}{ccc} \text{COV}\left[Z_{T},Z_{C}\right] & =Nr_{1}\left(-\left(tp\mu_{T_{+}}+(1-p)(1-s)\mu_{T_{-}}\right)\left((1-t)p\mu_{C_{+}}+s(1-p)\mu_{C_{-}}\right)-p_{⊕}p_{⊖}\mu_{T}\mu_{C}\right. & \\ & +\left.p_{⊖}\mu_{C}\left(tp\mu_{T_{+}}+(1-p)(1-s)\mu_{T_{-}}\right)+p_{⊕}\mu_{T}\left((1-t)p\mu_{C_{+}}+s(1-p)\mu_{C_{-}}\right)\right). \end{array}$$

The variances and covariances of *Z*_*T*_ and *Z*_*C*_ can be estimated as follows:

(C13) $$\begin{array}{ccc} \hat{\mathrm{VAR}}\left[Z_{T}\right] & =\frac{1}{Nr_{1}-1}\left(Nr_{1}\sum_{T_{⊕}}y^{2}-{\left(\sum_{T_{⊕}}y\right)}^{2}+Nr_{1}n_{T_{⊕}}\left(n_{T_{⊕}}-1\right)\frac{s_{T}^{2}}{n_{T}}\right. & \\ & \;\left.+Nr_{1}n_{T_{⊕}}\left(1-\frac{n_{T_{⊕}}}{Nr_{1}}\right){\left(\frac{1}{n_{T}}\sum_{T}y\right)}^{2}-2Nr_{1}\left(1-\frac{n_{T_{⊕}}}{Nr_{1}}\right)\left(\sum_{T_{⊕}}y\right)\left(\frac{1}{n_{T}}\sum_{T}y\right)\right) \end{array}$$

(C14) $$\begin{array}{ccc} \hat{\mathrm{VAR}}\left[Z_{C}\right] & =\frac{1}{Nr_{1}-1}\left(Nr_{1}\sum_{C_{⊖}}y^{2}-{\left(\sum_{C_{⊖}}y\right)}^{2}+Nr_{1}n_{C_{⊖}}\left(n_{C_{⊖}}-1\right)\frac{s_{C}^{2}}{n_{C}}\right. & \\ & \;\left.+Nr_{1}n_{C_{⊖}}\left(1-\frac{n_{C_{⊖}}}{Nr_{1}}\right){\left(\frac{1}{n_{C}}\sum_{C}y\right)}^{2}-2Nr_{1}\left(1-\frac{n_{C_{⊖}}}{Nr_{1}}\right)\left(\sum_{C_{⊖}}y\right)\left(\frac{1}{n_{C}}\sum_{C}y\right)\right) \end{array}$$

(C15) $$\begin{array}{ccc} \hat{\text{COV}}\left[Z_{T},Z_{C}\right] & =\frac{1}{Nr_{1}-1}\left(-\left(\sum_{T_{⊕}}y\right)\left(\sum_{C_{⊖}}y\right)+\left(\sum_{T_{⊕}}y\right)n_{C_{⊖}}\left(\frac{1}{n_{C}}\sum_{C}y\right)\right. & \\ & \;+\left.\left(\sum_{C_{⊖}}y\right)n_{T_{⊕}}\left(\frac{1}{n_{T}}\sum_{T}y\right)-n_{T_{⊕}}n_{C_{⊖}}\left(\frac{1}{n_{T}}\sum_{T}y\right)\left(\frac{1}{n_{C}}\sum_{C}y\right)\right) \end{array}$$

The derivations can be found in the Supporting Information. We anticipate the test statistics to approximately follow a normal distribution.

## APPENDIX D SIMULATION RESULTS FOR THE TYPE I ERROR AND POWER

### D.1

#### D.1.1

In order to verify the sample sizes obtained in Section 6, we simulated 10 000 data sets and estimated the resulting type I error and power (Table D1). The power was estimated using the same values as described in Sections 2 and 6. For the type I error, the means were set to $\mu_{T_{+}}=90$, $\mu_{T_{-}}=70$, $\mu_{C_{-}}=75$, and $\mu_{C_{-}}=55$ (ie, assuming that there is no interaction effect).

**Table D1:** Results for the power based on 10 000 simulations for different values of p, t, s, r_1_, and r_2_

			Interaction effect					Traditional analysis				
*p*	***t***	***s***	***r*** **_1_**	***r*** **_2_**	***N*_*I*_**	***α***	**1** − ***β***	***r***_1_	***r***_2_	***N*_*T**r**a**d*_**	***α***	1 − ***β***
0.15	0.8	0.8	0.50	0.29	1498	0.0499	0.7927	0.51	0.29	1580	0.0520	0.8211
0.15	0.9	0.9	0.50	0.22	836	0.0510	0.7938	0.50	0.22	853	0.0466	0.8083
0.15	1.0	1.0	0.49	0.15	530	0.0475	0.7877	0.49	0.15	516	0.0482	0.7884
0.20	0.8	0.8	0.50	0.32	974	0.0518	0.7995	0.51	0.32	1015	0.0473	0.8147
0.20	0.9	0.9	0.49	0.26	541	0.0467	0.7939	0.50	0.26	549	0.0461	0.7985
0.20	1.0	1.0	0.49	0.20	341	0.0486	0.7964	0.48	0.20	333	0.0462	0.7795
0.25	0.8	0.8	0.49	0.35	722	0.0500	0.7892	0.50	0.35	745	0.0489	0.8065
0.25	0.9	0.9	0.49	0.30	400	0.0489	0.7967	0.49	0.30	404	0.0473	0.7963
0.25	1.0	1.0	0.48	0.25	251	0.0498	0.7943	0.48	0.25	246	0.0490	0.7876
0.30	0.8	0.8	0.49	0.38	584	0.0478	0.7987	0.50	0.38	598	0.0470	0.8086
0.30	0.9	0.9	0.49	0.34	322	0.0451	0.7900	0.49	0.34	325	0.0533	0.7986
0.30	1.0	1.0	0.48	0.30	201	0.0490	0.7925	0.48	0.30	198	0.0500	0.7830
0.35	0.8	0.8	0.49	0.41	503	0.0462	0.7912	0.50	0.41	512	0.0503	0.8029
0.35	0.9	0.9	0.49	0.38	277	0.0480	0.7870	0.49	0.38	278	0.0473	0.7982
0.35	1.0	1.0	0.48	0.35	172	0.0468	0.7757	0.48	0.35	170	0.0473	0.7697
0.40	0.8	0.8	0.49	0.44	455	0.0478	0.7904	0.50	0.44	461	0.0506	0.7994
0.40	0.9	0.9	0.48	0.42	250	0.0497	0.7829	0.49	0.42	251	0.0496	0.7891
0.40	1.0	1.0	0.47	0.40	154	0.0477	0.7882	0.47	0.40	154	0.0500	0.7787
0.45	0.8	0.8	0.49	0.47	430	0.0476	0.7856	0.49	0.47	433	0.0471	0.8060
0.45	0.9	0.9	0.48	0.46	235	0.0479	0.7817	0.49	0.46	236	0.0488	0.7902
0.45	1.0	1.0	0.47	0.45	145	0.0460	0.7799	0.47	0.45	145	0.0445	0.7885
0.50	0.8	0.8	0.49	0.50	422	0.0493	0.7967	0.49	0.50	423	0.0489	0.7974
0.50	0.9	0.9	0.48	0.50	231	0.0485	0.7865	0.48	0.50	231	0.0455	0.7795
0.50	1.0	1.0	0.47	0.50	142	0.0396	0.7636	0.47	0.50	142	0.0436	0.7613
0.55	0.8	0.8	0.49	0.53	430	0.0463	0.7922	0.49	0.53	430	0.0494	0.7858
0.55	0.9	0.9	0.48	0.54	235	0.0449	0.7841	0.48	0.54	235	0.0460	0.7834
0.55	1.0	1.0	0.47	0.55	145	0.0460	0.7809	0.47	0.55	145	0.0447	0.7870
0.60	0.8	0.8	0.49	0.56	455	0.0482	0.7939	0.49	0.56	454	0.0511	0.7959
0.60	0.9	0.9	0.48	0.58	250	0.0484	0.7847	0.48	0.58	249	0.0467	0.7791
0.60	1.0	1.0	0.47	0.60	154	0.0492	0.7966	0.47	0.60	154	0.0471	0.7973
0.65	0.8	0.8	0.49	0.59	503	0.0498	0.7998	0.49	0.59	501	0.0521	0.8003
0.65	0.9	0.9	0.49	0.62	277	0.0459	0.7881	0.49	0.62	275	0.0430	0.7891
0.65	1.0	1.0	0.48	0.65	172	0.0412	0.7645	0.48	0.65	170	0.0390	0.7657
0.70	0.8	0.8	0.49	0.62	584	0.0510	0.7985	0.49	0.62	582	0.0488	0.7921
0.70	0.9	0.9	0.49	0.66	322	0.0465	0.7908	0.49	0.66	320	0.0492	0.7888
0.70	1.0	1.0	0.48	0.70	201	0.0528	0.7974	0.48	0.70	198	0.0452	0.7855
0.75	0.8	0.8	0.49	0.65	722	0.0481	0.7958	0.49	0.65	720	0.0496	0.7915
0.75	0.9	0.9	0.49	0.70	400	0.0496	0.7942	0.49	0.70	396	0.0466	0.7875
0.75	1.0	1.0	0.48	0.75	251	0.0567	0.8028	0.48	0.75	246	0.0524	0.7891
0.80	0.8	0.8	0.50	0.68	974	0.0476	0.8042	0.50	0.68	974	0.0501	0.8010
0.80	0.9	0.9	0.49	0.74	541	0.0453	0.7931	0.49	0.74	536	0.0496	0.7895
0.80	1.0	1.0	0.49	0.80	341	0.0524	0.7901	0.48	0.80	333	0.0475	0.7860
0.85	0.8	0.8	0.50	0.71	1498	0.0517	0.7920	0.50	0.71	1506	0.0509	0.8039
0.85	0.9	0.9	0.50	0.78	836	0.0505	0.7955	0.49	0.78	829	0.0558	0.7971
0.85	1.0	1.0	0.49	0.85	530	0.0481	0.7887	0.49	0.85	516	0.0475	0.7904
