# Supplementary material for: An alternative method to analyse the biomarker‐strategy design
Source: Stat Med. 2018 Sep 9;37(30):4636–51. doi: 10.1002/sim.7940 (PMC6492198; doi:10.1002/sim.7940)
Supplement: Supplementary file 1 — Supporting info item [file SIM-37-4636-s001.pdf]

Supplemental Material

“An alternative method to analyse the Biomarker-strategy design”

C. U. Kunz, T. Jaki, and N. Stallard

July 24, 2017

Contents

1 Derivations **3**

2 Traditional analysis **7**

3 Alternative analysis method **7**
- 3.1 Treatment effect 7
- 3.2 Biomarker and interaction effect 8
- 3.3 Covariance structure of the test statistics 9

4 Sample size calculation **12**
- 4.1 Treatment effect 12
- 4.2 Biomarker and interaction effect 12

5 Estimators for the variances of the test statistics **13**

6 Confidence intervals **16**

1 Derivations

In this section, we provide general derivations to be used in later sections. Numbers in brackets underneath mathematical expressions refer to the Equation where the part of the expression was derived.

Let a denote the number of patients who are BM positive and are classified as being positive, let b denote the number of patients who are BM positive but are classified as negative, let c denote the number of patients who are BM negative but classified as positive, and let $d = N - a - b - c$ denote the number of patients who are BM negative and classified as negative. Then, the probability of observing a specific combination of a, b, c, d is given by:

$$\binom{N}{a}\binom{N-a}{b}\binom{N-a-b}{c}(tp)^a((1-t)p)^b((1-s)(1-p))^c(s(1-p))^{N-a-b-c} \quad (1)$$

Now, Nr_1 patients will be randomised into the BM group of which $a + c$ patients will be classified as BM positive. Hence, we get

$$\begin{aligned}
\mathrm{E}[n_{T_\oplus}] =& \sum_{a=0}^{Nr_1}\sum_{b=0}^{Nr_1-a}\sum_{c=0}^{Nr_1-a-b}\binom{Nr_1}{a}\binom{Nr_1-a}{b}\binom{Nr_1-a-b}{c} \\
&(tp)^a((1-t)p)^b((1-s)(1-p))^c(s(1-p))^{N-a-b-c}(a+c) \\
=& Nr_1(tp + (1-s)(1-p)) = Nr_1 p_\oplus && (2) \\
\mathrm{VAR}[n_{T_\oplus}] =& Nr_1(tp + (1-s)(1-p))(1 - (tp + (1-s)(1-p))) = Nr_1 p_\oplus p_\ominus && (3) \\
\mathrm{E}[n_{C_\ominus}] =& \sum_{a=0}^{Nr_1}\sum_{b=0}^{Nr_1-a}\sum_{c=0}^{Nr_1-a-b}\binom{Nr_1}{a}\binom{Nr_1-a}{b}\binom{Nr_1-a-b}{c} \\
&(tp)^a((1-t)p)^b((1-s)(1-p))^c(s(1-p))^{N-a-b-c}(b+d) \\
=& Nr_1((1-t)p + s(1-p)) && (4) \\
\mathrm{VAR}[n_{C_\ominus}] =& Nr_1((1-t)p + s(1-p))(1 - ((1-t)p + s(1-p))) = Nr_1 p_\oplus p_\ominus && (5) \\
\mathrm{COV}[n_{T_\oplus}, n_{C_\ominus}] =& Nr_1((1-t)p + s(1-p))(1 - ((1-t)p + s(1-p))) = -Nr_1 p_\oplus p_\ominus && (6)
\end{aligned}$$

For the sums, we get:

$$\begin{aligned}
\mathrm{E}\left[\sum_{i=1}^{n_{T_\oplus}} y_{T_\oplus,i}\right] =& \sum_{a=0}^{Nr_1}\sum_{b=0}^{Nr_1-a}\sum_{c=0}^{Nr_1-a-b}\binom{Nr_1}{a}\binom{Nr_1-a}{b}\binom{Nr_1-a-b}{c} \\
&(tp)^a((1-t)p)^b((1-s)(1-p))^c(s(1-p))^{N-a-b-c}(a\mu_{T_+} + c\mu_{T_-}) \\
=& Nr_1(tp\mu_{T_+} + (1-s)(1-p)\mu_{T_-}) && (7) \\
\mathrm{VAR}\left[\sum_{i=1}^{n_{T_\oplus}} y_{T_\oplus,i}\right] =& Nr_1\left(pt(\mu_{T_+}^2 + \sigma_{T_+}^2) + (1-p)(1-s)(\mu_{T_-}^2 + \sigma_{T_-}^2) - (pt\mu_{T_+} + (1-p)(1-s)\mu_{T_-})^2\right) && (8) \\
\mathrm{E}\left[\sum_{i=1}^{n_{T_\oplus}} y_{T_\oplus,i}^2\right] =& \mathrm{E}\left[\sum_{i=1}^{n_{T_\oplus}} \mathrm{E}\left[y_{T_\oplus,i}^2\right]\right] = \mathrm{E}\left[\sum_{i=1}^{n_{T_\oplus}}\left(\mathrm{VAR}\left[y_{T_\oplus,i}\right] + \mathrm{E}\left[y_{T_\oplus,i}\right]^2\right)\right] \\
=& \mathrm{E}\left[\sum_{i=1}^{n_{T_\oplus}} \frac{pt}{p_\oplus}\left(\mu_{T_+}^2 + \sigma_{T_+}^2\right) + \frac{(1-p)(1-s)}{p_\oplus}\left(\mu_{T_-}^2 + \sigma_{T_-}^2\right) - \left(\frac{pt}{p_\oplus}\mu_{T_+} + \frac{(1-p)(1-s)}{p_\oplus}\mu_{T_-}\right)^2\right. \\
&\left. + \left(\frac{pt}{p_\oplus}\mu_{T_+} + \frac{(1-p)(1-s)}{p_\oplus}\mu_{T_-}\right)^2\right] \\
=& \mathrm{E}\left[\sum_{i=1}^{n_{T_\oplus}} \frac{1}{p_\oplus}\left(pt\left(\mu_{T_+}^2 + \sigma_{T_+}^2\right) + (1-p)(1-s)\left(\mu_{T_-}^2 + \sigma_{T_-}^2\right)\right)\right] \\
=& Nr_1\left(pt\left(\mu_{T_+}^2 + \sigma_{T_+}^2\right) + (1-p)(1-s)\left(\mu_{T_-}^2 + \sigma_{T_-}^2\right)\right) && (9)
\end{aligned}$$

$$\begin{aligned}
\mathrm{E}\left[\sum_{i=1}^{n_{C_\ominus}} y_{C_\ominus,i}\right] =& \sum_{a=0}^{Nr_1}\sum_{b=0}^{Nr_1-a}\sum_{c=0}^{Nr_1-a-b}\binom{Nr_1}{a}\binom{Nr_1-a}{b}\binom{Nr_1-a-b}{c}\\
&(tp)^a((1-t)p)^b((1-s)(1-p))^c(s(1-p))^{N-a-b-c}(b\mu_{C_+}+d\mu_{C_-})\\
=&Nr_1((1-t)p\mu_{C_+}+s(1-p)\mu_{C_-})
\end{aligned} \tag{10}$$

$$\mathrm{VAR}\left[\sum_{i=1}^{n_{C_\ominus}} y_{C_\ominus,i}\right] = Nr_1\left(p(1-t)(\mu_{C_+}^2+\sigma_{C_+}^2)+(1-p)s(\mu_{C_-}^2+\sigma_{C_-}^2)-(p(1-t)\mu_{C_+}+(1-p)s\mu_{C_-})^2\right) \tag{11}$$

$$\begin{aligned}
\mathrm{E}\left[\sum_{i=1}^{n_{C_\ominus}} y_{C_\ominus,i}^2\right] =& \mathrm{E}\left[\sum_{i=1}^{n_{C_\ominus}} \mathrm{E}\left[y_{C_\ominus,i}^2\right]\right] = \mathrm{E}\left[\sum_{i=1}^{n_{C_\ominus}}\left(\mathrm{VAR}\left[y_{C_\ominus,i}\right]+\mathrm{E}\left[y_{C_\ominus,i}\right]^2\right)\right]\\
=&\mathrm{E}\Bigg[\sum_{i=1}^{n_{C_\ominus}}\frac{p(1-t)}{p_\ominus}\left(\mu_{C_+}^2+\sigma_{C_+}^2\right)+\frac{(1-p)s}{p_\ominus}\left(\mu_{C_-}^2+\sigma_{C_-}^2\right)-\left(\frac{p(1-t)}{p_\ominus}\mu_{C_+}+\frac{(1-p)s}{p_\ominus}\mu_{C_-}\right)^2\\
&+\left(\frac{p(1-t)}{p_\ominus}\mu_{C_+}+\frac{(1-p)s}{p_\ominus}\mu_{C_-}\right)^2\Bigg]\\
&= \mathrm{E}\left[\sum_{i=1}^{n_{C_\ominus}}\frac{1}{p_\ominus}\left(p(1-t)\left(\mu_{C_+}^2+\sigma_{C_+}^2\right)+(1-p)s\left(\mu_{C_-}^2+\sigma_{C_-}^2\right)\right)\right]\\
&= Nr_1\left(p(1-t)\left(\mu_{C_+}^2+\sigma_{C_+}^2\right)+(1-p)s\left(\mu_{C_-}^2+\sigma_{C_-}^2\right)\right)
\end{aligned} \tag{12}$$

$$\mathrm{E}\left[\frac{1}{n_T}\sum_{i=1}^{n_T} y_{T,i}\right] = p\mu_{T_+}+(1-p)\mu_{T_-}=\mu_T \tag{13}$$

$$\mathrm{VAR}\left[\frac{1}{n_T}\sum_{i=1}^{n_T} y_{T,i}\right] = \frac{p(\mu_{T_+}^2+\sigma_{T_+}^2)+(1-p)(\mu_{T_-}^2+\sigma_{T_-}^2)-(p\mu_{T_+}+(1-p)\mu_{T_-})^2}{N(1-r_1)r_2} = \frac{\sigma_T^2}{N(1-r_1)r_2} \tag{14}$$

$$\mathrm{E}\left[\frac{1}{n_C}\sum_{i=1}^{n_C} y_{C,i}\right] = p\mu_{C_+}+(1-p)\mu_{C_-}=\mu_C \tag{15}$$

$$\mathrm{VAR}\left[\frac{1}{n_C}\sum_{i=1}^{n_C} y_{C,i}\right] = \frac{p(\mu_{C_+}^2+\sigma_{C_+}^2)+(1-p)(\mu_{C_-}^2+\sigma_{C_-}^2)-(p\mu_{C_+}+(1-p)\mu_{C_-})^2}{N(1-r_1)(1-r_2)} = \frac{\sigma_C^2}{N(1-r_1)(1-r_2)} \tag{16}$$

$$\begin{aligned}
\mathrm{E}\left[\frac{n_{T_\oplus}}{n_T}\sum_{i=1}^{n_T} y_{T,i}\right] =& \underbrace{\mathrm{E}\left[n_{T_\oplus}\right]}_{(2)}\underbrace{\mathrm{E}\left[\frac{1}{n_T}\sum_{i=1}^{n_T} y_{T,i}\right]}_{(13)}+\underbrace{\mathrm{COV}\left[n_{T_\oplus},\frac{1}{n_T}\sum_{i=1}^{n_T} y_{T,i}\right]}_{=0}\\
=&Nr_1(tp+(1-p)(1-s))\mu_T = Nr_1p_\oplus\mu_T
\end{aligned} \tag{17}$$

$$\begin{aligned}
\mathrm{VAR}\left[\frac{n_{T_\oplus}}{n_T}\sum_{i=1}^{n_T} y_{T,i}\right] =& \mathrm{E}\left[n_{T_\oplus}^2\right]\mathrm{E}\left[\left(\frac{1}{n_T}\sum_{i=1}^{n_T} y_{T,i}\right)^2\right]-\left(\mathrm{E}\left[n_{T_\oplus}\right]\right)^2\left(\mathrm{E}\left[\frac{1}{n_T}\sum_{i=1}^{n_T} y_{T,i}\right]\right)^2\\
=&\left(\underbrace{\left(\mathrm{E}\left[n_{T_\oplus}\right]\right)^2}_{(2)}+\underbrace{\mathrm{VAR}\left[n_{T_\oplus}\right]}_{(3)}\right)\left(\underbrace{\left(\mathrm{E}\left[\frac{1}{n_T}\sum_{i=1}^{n_T} y_{T,i}\right]\right)^2}_{(13)}+\underbrace{\mathrm{VAR}\left[\frac{1}{n_T}\sum_{i=1}^{n_T} y_{T,i}\right]}_{(14)}\right)\\
&-\underbrace{\left(\mathrm{E}\left[n_{T_\oplus}\right]\right)^2}_{(2)}\underbrace{\left(\mathrm{E}\left[\frac{1}{n_T}\sum_{i=1}^{n_T} y_{T,i}\right]\right)^2}_{(13)}\\
=&Nr_1p_\oplus(1-p_\oplus)\mu_T^2+((Nr_1p_\oplus)^2+Nr_1p_\oplus(1-p_\oplus))\frac{\sigma_T^2}{N(1-r_1)r_2}
\end{aligned} \tag{18}$$

$$\mathrm{E}\left[n_{C\ominus}\frac{1}{n_C}\sum_{i=1}^{n_C} y_{C,i}\right] = Nr_1 p_\ominus \mu_C \tag{19}$$

$$\mathrm{VAR}\left[n_{C\ominus}\frac{1}{n_C}\sum_{i=1}^{n_C} y_{C,i}\right] = Nr_1 p_\ominus(1-p_\ominus)\mu_C^2 + ((Nr_1 p_\ominus)^2 + Nr_1 p_\ominus(1-p_\ominus))\frac{\sigma_C^2}{N(1-r_1)(1-r_2)} \tag{20}$$

$$\begin{aligned}\mathrm{E}\left[\left(\sum_{i=1}^{n_{T\oplus}} y_{T_\oplus,i}\right) n_{T_\oplus}\right] =& \sum_{a=0}^{Nr_1}\sum_{b=0}^{Nr_1-a}\sum_{c=0}^{Nr_1-a-b}\binom{Nr_1}{a}\binom{Nr_1-a}{b}\binom{Nr_1-a-b}{c} \\ & (tp)^a((1-t)p)^b((1-s)(1-p))^c(s(1-p))^{N-a-b-c} \\ & (a\mu_{T_+} + c\mu_{T_-})(a+c) \\ =& Nr_1(1+Nr_1p_\oplus - p_\oplus)(tp\mu_{T_+} + (1-p)(1-s)\mu_{T_-})\end{aligned} \tag{21}$$

$$\mathrm{E}\left[\left(\sum_{i=1}^{n_{C\ominus}} y_{C_\ominus,i}\right) n_{C_\ominus}\right] = Nr_1(1+Nr_1p_\ominus - p_\ominus)((1-t)p\mu_{C_+} + (1-p)s\mu_{C_-}) \tag{22}$$

$$\mathrm{E}\left[\left(\sum_{i=1}^{n_{T\oplus}} y_{T_\oplus,i}\right) n_{C_\ominus}\right] = Nr_1(Nr_1-1)p_\ominus(tp\mu_{T_+} + (1-p)(1-s)\mu_{T_-}) \tag{23}$$

$$\mathrm{E}\left[\left(\sum_{i=1}^{n_{C\ominus}} y_{C_\ominus,i}\right) n_{T_\oplus}\right] = Nr_1(Nr_1-1)p_\oplus((1-t)p\mu_{C_+} + (1-p)s\mu_{C_-}) \tag{24}$$

$$\begin{aligned}\mathrm{E}\left[\left(\sum_{i=1}^{n_{T\oplus}} y_{T_\oplus +,i}\right)\left(\sum_{i=1}^{n_{C\ominus}} y_{C_\ominus,i}\right)\right] =& \sum_{a=0}^{Nr_1}\sum_{b=0}^{Nr_1-a}\sum_{c=0}^{Nr_1-a-b}\binom{Nr_1}{a}\binom{Nr_1-a}{b}\binom{Nr_1-a-b}{c} \\ & (tp)^a((1-t)p)^b((1-s)(1-p))^c(s(1-p))^{N-a-b-c} \\ & (a\mu_{T_+} + c\mu_{T_-})(b\mu_{C_+} + (Nr_1-a-b-c)\mu_{C_-}) \\ =& Nr_1(Nr_1-1)\left(tp\mu_{T_+} + (1-p)(1-s)\mu_{T_-}\right)\left(p(1-t)\mu_{C_+} + s(1-p)\mu_{C_-}\right)\end{aligned} \tag{25}$$

$$\begin{aligned}\mathrm{COV}\left[\sum_{i=1}^{n_{T\oplus}} y_{T_\oplus,i}, \frac{n_{T\oplus}}{n_T}\sum_{i=1}^{n_T} y_{T,i}\right] =& \mathrm{E}\left[\left(\sum_{i=1}^{n_{T\oplus}} y_{T_\oplus,i} - \mathrm{E}\left[\sum_{i=1}^{n_{T\oplus}} y_{T_\oplus,i}\right]\right)\left(n_{T_\oplus}\frac{1}{n_T}\sum_{i=1}^{n_T} y_{T,i} - \mathrm{E}\left[n_{T_\oplus}\frac{1}{n_T}\sum_{i=1}^{n_T} y_{T,i}\right]\right)\right] \\ =& \mathrm{E}\left[\left(\sum_{i=1}^{n_{T\oplus}} y_{T_\oplus,i}\right) n_{T_\oplus}\left(\frac{1}{n_T}\sum_{i=1}^{n_T} y_{T,i}\right)\right] - \mathrm{E}\left[\sum_{i=1}^{n_{T\oplus}} y_{T\oplus,i}\right]\mathrm{E}[n_{T_\oplus}]\mathrm{E}\left[\frac{1}{n_T}\sum_{i=1}^{n_T} y_{T,i}\right] \\ =& \underbrace{\mathrm{E}\left[\left(\sum_{i=1}^{n_{T\oplus}} y_{T\oplus,i}\right) n_{T_\oplus}\right]}_{(21)}\underbrace{\mathrm{E}\left[\frac{1}{n_T}\sum_{i=1}^{n_T} y_{T,i}\right]}_{(13)} - \underbrace{\mathrm{E}\left[\sum_{i=1}^{n_{T\oplus}} y_{T\oplus,i}\right]}_{(7)}\underbrace{\mathrm{E}[n_{T_\oplus}]}_{(2)}\underbrace{\mathrm{E}\left[\frac{1}{n_T}\sum_{i=1}^{n_T} y_{T,i}\right]}_{(13)} \\ =& Nr_1(1+Nr_1p_\oplus - p_\oplus)(tp\mu_{T_+} + (1-p)(1-s)\mu_{T_-})\mu_T \\ & - Nr_1(tp\mu_{T_+} + (1-p)(1-s)\mu_{T_-})Nr_1p_\oplus\mu_T \\ =& Nr_1\mu_T(tp\mu_{T_+} + (1-p)(1-s)\mu_{T_-})(1-p_\oplus)\end{aligned} \tag{26}$$

$$\begin{aligned}\mathrm{COV}\left[\sum_{i=1}^{n_{C\ominus}} y_{C_\ominus,i}, \frac{n_{C\ominus}}{n_C}\sum_{i=1}^{n_C} y_{C,i}\right] =& \mathrm{E}\left[\left(\sum_{i=1}^{n_{C\ominus}} y_{C_\ominus,i} - \mathrm{E}\left[\sum_{i=1}^{n_{C\ominus}} y_{C_\ominus,i}\right]\right)\left(n_{C_\ominus}\frac{1}{n_C}\sum_{i=1}^{n_C} y_{C,i} - \mathrm{E}\left[n_{C_\ominus}\frac{1}{n_C}\sum_{i=1}^{n_C} y_{C,i}\right]\right)\right] \\ =& \mathrm{E}\left[\left(\sum_{i=1}^{n_{C\ominus}} y_{C_\ominus,i}\right) n_{C_\ominus}\left(\frac{1}{n_C}\sum_{i=1}^{n_C} y_{C,i}\right)\right] - \mathrm{E}\left[\sum_{i=1}^{n_{C\ominus}} y_{C_\ominus,i}\right]\mathrm{E}[n_{C_\ominus}]\mathrm{E}\left[\frac{1}{n_C}\sum_{i=1}^{n_C} y_{C,i}\right] \\ =& \underbrace{\mathrm{E}\left[\left(\sum_{i=1}^{n_{C\ominus}} y_{C_\ominus,i}\right) n_{C_\ominus}\right]}_{(22)}\underbrace{\mathrm{E}\left[\frac{1}{n_C}\sum_{i=1}^{n_C} y_{C,i}\right]}_{(15)} - \underbrace{\mathrm{E}\left[\sum_{i=1}^{n_{C\ominus}} y_{C_\ominus,i}\right]}_{(10)}\underbrace{\mathrm{E}[n_{C_\ominus}]}_{(4)}\underbrace{\mathrm{E}\left[\frac{1}{n_C}\sum_{i=1}^{n_C} y_{C,i}\right]}_{(15)} \\ =& Nr_1(1+Nr_1p_\ominus - p_\ominus)((1-t)p\mu_{C_-} + (1-p)(1-s)\mu_{C_-})\mu_C \\ & - Nr_1((1-t)p\mu_{C_-} + (1-p)(1-s)\mu_{C_-})Nr_1p_\ominus\mu_C \\ =& Nr_1\mu_C((1-t)p\mu_{C_+} + (1-p)s\mu_{C_-})(1-p_\ominus)\end{aligned} \tag{27}$$

$$\mathrm{COV}\left[\sum_{i=1}^{n_{T_\oplus}} y_{T_\oplus,i}, \sum_{i=1}^{n_{C_\ominus}} y_{C_\ominus,i}\right] = \underbrace{\mathrm{E}\left[\left(\sum_{i=1}^{n_{T_\oplus}} y_{T_\oplus,i}\right)\left(\sum_{i=1}^{n_{C_\ominus}} y_{C_\ominus,i}\right)\right]}_{(25)} - \underbrace{\mathrm{E}\left[\sum_{i=1}^{n_{T_\oplus}} y_{T_\oplus,i}\right]}_{(7)}\underbrace{\mathrm{E}\left[\sum_{i=1}^{n_{C_\ominus}} y_{C_\ominus,i}\right]}_{(10)}$$

$$= -Nr_1\left(tp\mu_{T_+} + (1-p)(1-s)\mu_{T_-}\right)\left((1-t)p\mu_{C_+} + s(1-p)\mu_{C_-}\right) \quad (28)$$

$$\mathrm{COV}\left[\sum_{i=1}^{n_{T_\oplus}} y_{T_\oplus,i}, \frac{n_{C_\ominus}}{n_C}\sum_{i=1}^{n_C} y_{C,i}\right] = \underbrace{\mathrm{E}\left[\left(\sum_{i=1}^{n_{T_\oplus}} y_{T_\oplus,i}\right) n_{C_\ominus}\right]}_{(23)}\underbrace{\mathrm{E}\left[\frac{1}{n_C}\sum_{i=1}^{n_C} y_{C,i}\right]}_{(15)} - \underbrace{\mathrm{E}\left[\sum_{i=1}^{n_{T_\oplus}} y_{T_\oplus,i}\right]}_{(7)}\underbrace{\mathrm{E}\left[\frac{n_{C_\ominus}}{n_C}\sum_{i=1}^{n_C} y_{C,i}\right]}_{(19)}$$

$$= -Nr_1 p_\ominus \mu_C (tp\mu_{T_+} + (1-p)(1-s)\mu_{T_-}) \quad (29)$$

$$\mathrm{COV}\left[\sum_{i=1}^{n_{C_\ominus}} y_{C_\ominus,i}, \frac{n_{T_\oplus}}{n_T}\sum_{i=1}^{n_T} y_{T,i}\right] = -Nr_1 p_\oplus \mu_T ((1-t)p\mu_{C_+} + s(1-p)\mu_{C_-}) \quad (30)$$

$$\begin{aligned}
\mathrm{COV}\left[\frac{n_{T_\oplus}}{n_T}\sum_{i=1}^{n_T} y_{T,i}, \frac{n_{C_\ominus}}{n_C}\sum_{i=1}^{n_C} y_{C,i}\right] =& \mathrm{E}\left[\frac{n_{T_\oplus}}{n_T}\sum_{i=1}^{n_T} y_{T,i}\frac{n_{C_\oplus}}{n_C}\sum_{i=1}^{n_C} y_{C,i}\right] - \mathrm{E}\left[\frac{n_{T_\oplus}}{n_T}\sum_{i=1}^{n_T} y_{T,i}\right]\mathrm{E}\left[\frac{n_{C_\ominus}}{n_C}\sum_{i=1}^{n_C} y_{C,i}\right] \\
=& \mathrm{E}\left[n_{T_\oplus} n_{C_\ominus}\right]\mathrm{E}\left[\frac{1}{n_T}\sum_{i=1}^{n_T} y_{T,i}\frac{1}{n_C}\sum_{i=1}^{n_C} y_{C,i}\right] \\
&- \mathrm{E}\left[\frac{n_{T_\oplus}}{n_T}\sum_{i=1}^{n_T} y_{T,i}\right]\mathrm{E}\left[\frac{n_{C_\ominus}}{n_C}\sum_{i=1}^{n_C} y_{C,i}\right] \\
=& \left(\underbrace{\mathrm{E}\left[n_{T_\oplus}\right]}_{(2)}\underbrace{\mathrm{E}\left[n_{C_\ominus}\right]}_{(4)} + \underbrace{\mathrm{COV}\left[n_{T_\oplus}, n_{C_\ominus}\right]}_{(6)}\right)\underbrace{\mathrm{E}\left[\frac{1}{n_T}\sum_{i=1}^{n_T} y_{T,i}\right]}_{(13)}\underbrace{\mathrm{E}\left[\frac{1}{n_C}\sum_{i=1}^{n_C} y_{C,i}\right]}_{(15)} \\
&- \underbrace{\mathrm{E}\left[\frac{n_{T_\oplus}}{n_T}\sum_{i=1}^{n_T} y_{T,i}\right]}_{(17)}\underbrace{\mathrm{E}\left[\frac{n_{C_\ominus}}{n_C}\sum_{i=1}^{n_C} y_{C,i}\right]}_{(19)} \quad (31)
\end{aligned}$$

2 Traditional analysis

$$\begin{aligned}
\mathrm{E}\left[Z_{BM}-Z_R\right]=&\mathrm{E}\left[\frac{1}{Nr_1}\left(\sum_{i=1}^{n_{T\oplus}} y_{T\oplus,i}+\sum_{i=1}^{n_{C\ominus}} y_{C\ominus,i}\right)\right]-\mathrm{E}\left[\frac{1}{N(1-r_1)}\left(\sum_{i=1}^{n_T} y_{T,i}+\sum_{i=1}^{n_C} y_{C,i}\right)\right]\\
=&\frac{1}{Nr_1}\underbrace{\mathrm{E}\left[\sum_{i=1}^{n_{T\oplus}} y_{T\oplus,i}\right]}_{(7)}+\frac{1}{Nr_1}\underbrace{\mathrm{E}\left[\sum_{i=1}^{n_{C\ominus}} y_{C\ominus,i}\right]}_{(10)}\\
&-\frac{N(1-r_1)r_2}{N(1-r_1)}\underbrace{\mathrm{E}\left[\frac{1}{n_T}\sum_{i=1}^{n_T} y_{T,i}\right]}_{(13)}-\frac{N(1-r_1)(1-r_2)}{N(1-r_1)}\underbrace{\mathrm{E}\left[\frac{1}{n_C}\sum_{i=1}^{n_C} y_{C,i}\right]}_{(15)} \qquad (32)
\end{aligned}$$

$$\begin{aligned}
\mathrm{VAR}\left[Z_{BM}-Z_R\right]=&\mathrm{VAR}\left[Z_{BM}\right]+\mathrm{VAR}\left[Z_R\right]-2\mathrm{COV}\left[Z_{BM},Z_R\right]\\
=&\frac{1}{Nr_1}\underbrace{\mathrm{VAR}\left[\sum_{i=1}^{n_{T\oplus}} y_{T\oplus,i}\right]}_{(8)}+\frac{1}{Nr_1}\underbrace{\mathrm{VAR}\left[\sum_{i=1}^{n_{C\ominus}} y_{C\ominus,i}\right]}_{(11)}+2\frac{1}{(Nr_1)^2}\underbrace{\mathrm{COV}\left[\sum_{i=1}^{n_{T\oplus}} y_{T\oplus,i},\sum_{i=1}^{n_{C\ominus}} y_{C\ominus,i}\right]}_{(28)}\\
&+\frac{(N(1-r_1)r_2)^2}{N(1-r_1)}\underbrace{\mathrm{VAR}\left[\frac{1}{n_T}\sum_{i=1}^{n_T} y_{T,i}\right]}_{(14)}+\frac{(N(1-r_1)(1-r_2))^2}{N(1-r_1)}\underbrace{\mathrm{VAR}\left[\frac{1}{n_C}\sum_{i=1}^{n_C} y_{C,i}\right]}_{(16)}\\
&+2\frac{1}{(N(1-r_1))^2}\underbrace{\mathrm{COV}\left[\sum_{i=1}^{n_T} y_{T,i},\sum_{i=1}^{n_C} y_{C,i}\right]}_{=0}\\
&-2\frac{1}{N^2r_1(1-r_1)}\underbrace{\mathrm{COV}\left[\sum_{i=1}^{n_{T\oplus}} y_{T\oplus,i}+\sum_{i=1}^{n_{C\ominus}} y_{C\ominus,i},\sum_{i=1}^{n_T} y_{T,i}+\sum_{i=1}^{n_C} y_{C,i}\right]}_{=0} \qquad (33)
\end{aligned}$$

3 Alternative analysis method

3.1 Treatment effect

The expected value and variance for the test statistic for the treatment effect is given by (see Equations (29) and (30) in the main article):

$$\begin{aligned}
\mathrm{E}\left[Z_{TR}-Z_{CR}\right]=&\underbrace{\mathrm{E}\left[\frac{1}{n_T}\sum_{i=1}^{n_T} y_{T,i}\right]}_{(13)}-\underbrace{\mathrm{E}\left[\frac{1}{n_C}\sum_{i=1}^{n_C} y_{C,i}\right]}_{(15)}=p\left(\mu_{T_+}-\mu_{C_+}\right)+(1-p)\left(\mu_{T_-}-\mu_{C_-}\right)\\
=&p\mu_{T_+}+(1-p)\mu_{T_-}-\left(p\mu_{C_+}+(1-p)\mu_{C_-}\right)=\mu_T-\mu_C \qquad (34)
\end{aligned}$$

$$\begin{aligned}
\mathrm{VAR}\left[Z_{TR}-Z_{CR}\right]=&\underbrace{\mathrm{VAR}\left[\frac{1}{n_T}\sum_{i=1}^{n_T} y_{T,i}\right]}_{(14)}+\underbrace{\mathrm{VAR}\left[\frac{1}{n_C}\sum_{i=1}^{n_C} y_{C,i}\right]}_{(16)}-2\underbrace{\mathrm{COV}\left[\frac{1}{n_T}\sum_{i=1}^{n_T} y_{T,i},\frac{1}{n_C}\sum_{i=1}^{n_C} y_{C,i}\right]}_{=0}\\
=&\frac{p(\mu_{T_+}^2+\sigma_{T_+}^2)+(1-p)(\mu_{T_-}^2+\sigma_{T_-}^2)-(p\mu_{T_+}+(1-p)\mu_{T_-})^2}{N(1-r_1)r_2}\\
&+\frac{p(\mu_{C_+}^2+\sigma_{C_+}^2)+(1-p)(\mu_{C_-}^2+\sigma_{C_-}^2)-(p\mu_{C_+}+(1-p)\mu_{C_-})^2}{N(1-r_1)(1-r_2)}\\
=&\frac{\sigma_T^2}{N\left(1-r_1\right)r_2}+\frac{\sigma_C^2}{N\left(1-r_1\right)\left(1-r_2\right)}. \qquad (35)
\end{aligned}$$

3.2 Biomarker and interaction effect

Let Z_T and Z_C be defined as follows (see Equations (8) and (9) in main article):

$$Z_T = \sum_{i=1}^{n_{T_\oplus}} y_{T_\oplus,i} - \frac{n_{T_\oplus}}{n_T}\sum_{i=1}^{n_T} y_{T,i} \tag{36}$$

$$Z_C = \sum_{i=1}^{n_{C_\ominus}} y_{C_\ominus,i} - \frac{n_{C_\ominus}}{n_C}\sum_{i=1}^{n_C} y_{C,i}. \tag{37}$$

The expected values are given by:

$$\begin{aligned}
\mathrm{E}[Z_T] =& \mathrm{E}\left[\sum_{i=1}^{n_{T_\oplus}} y_{T_\oplus,i} - \frac{n_{T_\oplus}}{n_T}\sum_{i=1}^{n_T} y_{T,i}\right] = \underbrace{\mathrm{E}\left[\sum_{i=1}^{n_{T_\oplus}} y_{T_\oplus,i}\right]}_{(7)} - \underbrace{\mathrm{E}\left[\frac{n_{T_\oplus}}{n_T}\sum_{i=1}^{n_T} y_{T,i}\right]}_{(17)}\\
=& Nr_1(tp\mu_{T_+} + (1-p)(1-s)\mu_{T_-}) - Nr_1(tp + (1-p)(1-s))\mu_T\\
=& Nr_1 p(1-p)(1-s-t)\left(\mu_{T_-} - \mu_{T_+}\right) \qquad (38)\\
\mathrm{E}[Z_C] =& \mathrm{E}\left[\sum_{i=1}^{n_{C_\ominus}} y_{C_\ominus,i} - \frac{n_{C_\ominus}}{n_C}\sum_{i=1}^{n_C} y_{C,i}\right] = \underbrace{\mathrm{E}\left[\sum_{i=1}^{n_{C_\ominus}} y_{C_\ominus,i}\right]}_{(10)} - \underbrace{\mathrm{E}\left[\frac{n_{C_\ominus}}{n_C}\sum_{i=1}^{n_C} y_{C,i}\right]}_{(19)}\\
=& Nr_1((1-t)p\mu_{C_+} + s(1-p)\mu_{C_-}) - Nr_1(p(1-t) + s(1-p))\mu_C\\
=& Nr_1 p(1-p)(1-s-t)\left(\mu_{C_+} - \mu_{C_-}\right) \qquad (39)
\end{aligned}$$

The variances are given by:

$$\begin{aligned}
\mathrm{VAR}[Z_T] =& \mathrm{VAR}\left[\sum_{i=1}^{n_{T_\oplus}} y_{T_\oplus,i} - \frac{n_{T_\oplus}}{n_T}\sum_{i=1}^{n_T} y_{T,i}\right]\\
=& \underbrace{\mathrm{VAR}\left[\sum_{i=1}^{n_{T_\oplus}} y_{T_\oplus,i}\right]}_{(8)} + \underbrace{\mathrm{VAR}\left[\frac{n_{T_\oplus}}{n_T}\sum_{i=1}^{n_T} y_{T,i}\right]}_{(18)} \underbrace{-2\,\mathrm{COV}\left[\sum_{i=1}^{n_{T_\oplus}} y_{T_\oplus,i}, \frac{n_{T_\oplus}}{n_T}\sum_{i=1}^{n_T} y_{T,i}\right]}_{(26)}\\
=& Nr_1\left(pt(\mu_{T_+}^2 + \sigma_{T_+}^2) + (1-p)(1-s)(\mu_{T_-}^2 + \sigma_{T_-}^2) - (pt\mu_{T_+} + (1-p)(1-s)\mu_{T_-})^2\right)\\
&+ Nr_1 p_\oplus(1-p_\oplus)\mu_T^2 + ((Nr_1 p_\oplus)^2 + Nr_1 p_\oplus(1-p_\oplus))\frac{\sigma_T^2}{N(1-r_1)r_2}\\
&- 2\left(Nr_1\mu_T(tp\mu_{T_+} + (1-p)(1-s)\mu_{T_-})(1-p_\oplus)\right) \qquad (40)\\
\mathrm{VAR}[Z_C] =& \mathrm{VAR}\left[\sum_{i=1}^{n_{C_\ominus}} y_{C_\ominus,i} - \frac{n_{C_\ominus}}{n_C}\sum_{i=1}^{n_C} y_{C,i}\right]\\
=& \underbrace{\mathrm{VAR}\left[\sum_{i=1}^{n_{C_\ominus}} y_{C_\ominus,i}\right]}_{(11)} + \underbrace{\mathrm{VAR}\left[\frac{n_{C_\ominus}}{n_C}\sum_{i=1}^{n_C} y_{C,i}\right]}_{(20)} \underbrace{-2\,\mathrm{COV}\left[\sum_{i=1}^{n_{C_\ominus}} y_{C_\ominus,i}, \frac{n_{C_\ominus}}{n_C}\sum_{i=1}^{n_C} y_{C,i}\right]}_{(27)}\\
=& Nr_1\left(p(1-t)(\mu_{C_+}^2 + \sigma_{C_+}^2) + (1-p)s(\mu_{C_-}^2 + \sigma_{C_-}^2) - (p(1-t)\mu_{C_+} + (1-p)s\mu_{C_-})^2\right)\\
&+ Nr_1 p_\ominus(1-p_\ominus)\mu_C^2 + ((Nr_1 p_\ominus)^2 + Nr_1 p_\ominus(1-p_\ominus))\frac{\sigma_C^2}{N(1-r_1)(1-r_2)}\\
&- 2\left(Nr_1\mu_C((1-t)p\mu_{C_+} + (1-p)s\mu_{C_-})(1-p_\ominus)\right) \qquad (41)
\end{aligned}$$

and the covariance by:

$$\mathrm{COV}\left[Z_T, Z_C\right] = \underbrace{\mathrm{COV}\left[\sum_{i=1}^{n_{T_\oplus}} y_{T_\oplus,i}, \sum_{i=1}^{n_{C_\ominus}} y_{C_\ominus,i}\right]}_{(28)} - \underbrace{\mathrm{COV}\left[\sum_{i=1}^{n_{T_\oplus}} y_{T_\oplus,i}, \frac{n_{C_\ominus}}{n_C}\sum_{i=1}^{n_C} y_{C,i}\right]}_{(29)}$$
$$- \underbrace{\mathrm{COV}\left[\sum_{i=1}^{n_{C_\ominus}} y_{C_\ominus,i}, \frac{n_{T_\oplus}}{n_T}\sum_{i=1}^{n_T} y_{n_T,i}\right]}_{(30)} + \underbrace{\mathrm{COV}\left[\frac{n_{T_\oplus}}{n_T}\sum_{i=1}^{n_T} y_{n_T,i}, \frac{n_{C_\ominus}}{n_C}\sum_{i=1}^{n_C} y_{C,i}\right]}_{(31)} \tag{42}$$

3.3 Covariance structure of the test statistics

$$\begin{aligned}
\mathrm{COV}_{T,B} =& \mathrm{COV}\left[T_T, T_B\right] = \mathrm{COV}\left[\frac{Z_{TR}-Z_{CR}}{\sqrt{\underbrace{\mathrm{VAR}\left[Z_{TR}-Z_{CR}\right]}_{(35)}}}, \frac{Z_T - Z_C}{\sqrt{\mathrm{VAR}\left[Z_T - Z_C\right]}}\right] \\
=& \frac{\mathrm{COV}\left[Z_{TR}-Z_{CR}, Z_T - Z_C\right]}{\sqrt{\mathrm{VAR}\left[Z_{TR}-Z_{CR}\right]}\sqrt{\mathrm{VAR}\left[Z_T-Z_C\right]}} \\
=& \frac{\mathrm{COV}\left[\frac{1}{n_T}\sum_{i=1}^{n_T} y_{i,T} - \frac{1}{n_C}\sum_{i=1}^{n_C} y_{i,C}, \frac{1}{n_{T_\oplus}}\sum_{i=1}^{n_{T_\oplus}} y_{i,T_\oplus} - \frac{n_{T_\oplus}}{n_T}\sum_{i=1}^{n_T} y_{i,T} - \frac{1}{n_{C_\ominus}}\sum_{i=1}^{n_{C_\ominus}} y_{i,C_\ominus} + \frac{n_{C_\ominus}}{n_C}\sum_{i=1}^{n_C} y_{i,C}\right]}{\sqrt{\frac{r_2\sigma_C^2+(1-r_2)\sigma_T^2}{N(1-r_1)r_2(1-r_2)}\left(\mathrm{VAR}\left[Z_T\right]+\mathrm{VAR}\left[Z_C\right]-2\mathrm{COV}\left[Z_T,Z_C\right]\right)}} \\
=& \frac{1}{\sqrt{\frac{r_2\sigma_C^2+(1-r_2)\sigma_T^2}{N(1-r_1)r_2(1-r_2)}\left(\mathrm{VAR}\left[Z_T\right]+\mathrm{VAR}\left[Z_C\right]-2\mathrm{COV}\left[Z_T,Z_C\right]\right)}} \\
&\left(\underbrace{\mathrm{COV}\left[\frac{1}{n_T}\sum_{i=1}^{n_T} y_{i,T}, \frac{1}{n_{T_\oplus}}\sum_{i=1}^{n_{T_\oplus}} y_{i,T_\oplus}\right]}_{=0} - \mathrm{COV}\left[\frac{1}{n_T}\sum_{i=1}^{n_T} y_{i,T}, \frac{n_{T_\oplus}}{n_T}\sum_{i=1}^{n_T} y_{i,T}\right]\right. \\
&- \underbrace{\mathrm{COV}\left[\frac{1}{n_T}\sum_{i=1}^{n_T} y_{i,T}, \frac{1}{n_{C_\ominus}}\sum_{i=1}^{n_{C_\ominus}} y_{i,C_\ominus}\right]}_{=0} + \underbrace{\mathrm{COV}\left[\frac{1}{n_T}\sum_{i=1}^{n_T} y_{i,T}, \frac{n_{C_\ominus}}{n_C}\sum_{i=1}^{n_C} y_{i,C}\right]}_{=0} \\
&- \underbrace{\mathrm{COV}\left[\frac{1}{n_C}\sum_{i=1}^{n_C} y_{i,C}, \frac{1}{n_{T_\oplus}}\sum_{i=1}^{n_{T_\oplus}} y_{i,T_\oplus}\right]}_{=0} + \underbrace{\mathrm{COV}\left[\frac{1}{n_C}\sum_{i=1}^{n_C} y_{i,C}, \frac{n_{T_\oplus}}{n_T}\sum_{i=1}^{n_T} y_{i,T}\right]}_{=0} \\
&\left.+ \underbrace{\mathrm{COV}\left[\frac{1}{n_C}\sum_{i=1}^{n_C} y_{i,C}, \frac{1}{n_{C_\ominus}}\sum_{i=1}^{n_{C_\ominus}} y_{i,C_\ominus}\right]}_{=0} - \mathrm{COV}\left[\frac{1}{n_C}\sum_{i=1}^{n_C} y_{i,C}, \frac{n_{C_\ominus}}{n_C}\sum_{i=1}^{n_C} y_{i,C}\right]\right) \\
=& \frac{-\mathrm{COV}\left[\frac{1}{n_T}\sum_{i=1}^{n_T} y_{i,T}, \frac{n_{T_\oplus}}{n_T}\sum_{i=1}^{n_T} y_{i,T}\right] - \mathrm{COV}\left[\frac{1}{n_C}\sum_{i=1}^{n_C} y_{i,C}, \frac{n_{C_\ominus}}{n_C}\sum_{i=1}^{n_C} y_{i,C}\right]}{\sqrt{\frac{r_2\sigma_C^2+(1-r_2)\sigma_T^2}{N(1-r_1)r_2(1-r_2)}\left(\mathrm{VAR}\left[Z_T\right]+\mathrm{VAR}\left[Z_C\right]-2\mathrm{COV}\left[Z_T,Z_C\right]\right)}} \\
=& \frac{-\left(\mathrm{E}\left[n_{T_\oplus}\left(\frac{1}{n_T}\sum_{i=1}^{n_T} y_{i,T}\right)^2\right] - \mathrm{E}\left[\frac{1}{n_T}\sum_{i=1}^{n_T} y_{i,T}\right]\mathrm{E}\left[n_{T_\oplus}\frac{1}{n_T}\sum_{i=1}^{n_T} y_{i,T}\right]\right)}{\sqrt{\frac{r_2\sigma_C^2+(1-r_2)\sigma_T^2}{N(1-r_1)r_2(1-r_2)}\left(\mathrm{VAR}\left[Z_T\right]+\mathrm{VAR}\left[Z_C\right]-2\mathrm{COV}\left[Z_T,Z_C\right]\right)}} \\
&+ \frac{-\left(\mathrm{E}\left[n_{C_\ominus}\left(\frac{1}{n_C}\sum_{i=1}^{n_C} y_{i,C}\right)^2\right] - \mathrm{E}\left[\frac{1}{n_C}\sum_{i=1}^{n_C} y_{i,C}\right]\mathrm{E}\left[n_{C_\ominus}\frac{1}{n_C}\sum_{i=1}^{n_C} y_{i,C}\right]\right)}{\sqrt{\frac{r_2\sigma_C^2+(1-r_2)\sigma_T^2}{N(1-r_1)r_2(1-r_2)}\left(\mathrm{VAR}\left[Z_T\right]+\mathrm{VAR}\left[Z_C\right]-2\mathrm{COV}\left[Z_T,Z_C\right]\right)}}
\end{aligned}$$

$$
\begin{aligned}
&= \frac{-\left(\mathrm{E}\left[n_{T_\oplus}\right]\mathrm{E}\left[\left(\frac{1}{n_T}\sum_{i=1}^{n_T} y_{i,T}\right)^2\right] - \mathrm{E}\left[n_{T_\oplus}\right]\mathrm{E}\left[\frac{1}{n_T}\sum_{i=1}^{n_T} y_{i,T}\right]^2\right)}{\sqrt{\frac{r_2\sigma_C^2+(1-r_2)\sigma_T^2}{N(1-r_1)r_2(1-r_2)}\left(\mathrm{VAR}\left[Z_T\right]+\mathrm{VAR}\left[Z_C\right]-2\mathrm{COV}\left[Z_T,Z_C\right]\right)}} \\
&+ \frac{-\left(\mathrm{E}\left[n_{C_\ominus}\right]\mathrm{E}\left[\left(\frac{1}{n_C}\sum_{i=1}^{n_C} y_{i,C}\right)^2\right] - \mathrm{E}\left[n_{C_\ominus}\right]\mathrm{E}\left[\frac{1}{n_C}\sum_{i=1}^{n_C} y_{i,C}\right]^2\right)}{\sqrt{\frac{r_2\sigma_C^2+(1-r_2)\sigma_T^2}{N(1-r_1)r_2(1-r_2)}\left(\mathrm{VAR}\left[Z_T\right]+\mathrm{VAR}\left[Z_C\right]-2\mathrm{COV}\left[Z_T,Z_C\right]\right)}} \\
&= \frac{-\left(\mathrm{E}\left[n_{T_\oplus}\right]\left(\mathrm{V}\left[\frac{1}{n_T}\sum_{i=1}^{n_T} y_{i,T}\right]+\mathrm{E}\left[\frac{1}{n_T}\sum_{i=1}^{n_T} y_{i,T}\right]^2\right) - \mathrm{E}\left[n_{T_\oplus}\right]\mathrm{E}\left[\frac{1}{n_T}\sum_{i=1}^{n_T} y_{i,T}\right]^2\right)}{\sqrt{\frac{r_2\sigma_C^2+(1-r_2)\sigma_T^2}{N(1-r_1)r_2(1-r_2)}\left(\mathrm{VAR}\left[Z_T\right]+\mathrm{VAR}\left[Z_C\right]-2\mathrm{COV}\left[Z_T,Z_C\right]\right)}} \\
&+ \frac{-\left(\mathrm{E}\left[n_{C_\ominus}\right]\left(\mathrm{V}\left[\frac{1}{n_C}\sum_{i=1}^{n_C} y_{i,C}\right]+\mathrm{E}\left[\frac{1}{n_C}\sum_{i=1}^{n_C} y_{i,C}\right]\right) - \mathrm{E}\left[n_{C_\ominus}\right]\mathrm{E}\left[\frac{1}{n_C}\sum_{i=1}^{n_C} y_{i,C}\right]^2\right)}{\sqrt{\frac{r_2\sigma_C^2+(1-r_2)\sigma_T^2}{N(1-r_1)r_2(1-r_2)}\left(\mathrm{VAR}\left[Z_T\right]+\mathrm{VAR}\left[Z_C\right]-2\mathrm{COV}\left[Z_T,Z_C\right]\right)}} \\
&= \frac{-\overbrace{\mathrm{E}\left[n_{T_\oplus}\right]}^{(2)}\overbrace{\mathrm{V}\left[\frac{1}{n_T}\sum_{i=1}^{n_T} y_{i,T}\right]}^{(14)} - \overbrace{\mathrm{E}\left[n_{C_\ominus}\right]}^{(4)}\overbrace{\mathrm{V}\left[\frac{1}{n_C}\sum_{i=1}^{n_C} y_{i,C}\right]}^{(16)}}{\sqrt{\frac{r_2\sigma_C^2+(1-r_2)\sigma_T^2}{N(1-r_1)r_2(1-r_2)}\left(\underbrace{\mathrm{VAR}\left[Z_T\right]}_{(40)}+\underbrace{\mathrm{VAR}\left[Z_C\right]}_{(41)}-2\underbrace{\mathrm{COV}\left[Z_T,Z_C\right]}_{(42)}\right)}}
\end{aligned}
\tag{43}
$$

$$
\begin{aligned}
\mathrm{COV}_{T,I} =& \mathrm{COV}\left[T_T,T_I\right] = \mathrm{COV}\left[\frac{Z_{TR}-Z_{CR}}{\sqrt{\underbrace{\mathrm{VAR}\left[Z_{TR}-Z_{CR}\right]}_{(35)}}}, \frac{Z_T+Z_C}{\sqrt{\mathrm{VAR}\left[Z_T+Z_C\right]}}\right] \\
=& \frac{\mathrm{COV}\left[Z_{TR}-Z_{CR}, Z_T+Z_C\right]}{\sqrt{\mathrm{VAR}\left[Z_{TR}-Z_{CR}\right]}\sqrt{\mathrm{VAR}\left[Z_T+Z_C\right]}} \\
=& \frac{\mathrm{COV}\left[\frac{1}{n_T}\sum_{i=1}^{n_T} y_{i,T} - \frac{1}{n_C}\sum_{i=1}^{n_C} y_{i,C}, \frac{1}{n_{T_\oplus}}\sum_{i=1}^{n_{T_\oplus}} y_{i,T_\oplus} - \frac{n_{T_\oplus}}{n_T}\sum_{i=1}^{n_T} y_{i,T} + \frac{1}{n_{C_\ominus}}\sum_{i=1}^{n_{C_\ominus}} y_{i,C_\ominus} - \frac{n_{C_\ominus}}{n_C}\sum_{i=1}^{n_C} y_{i,C}\right]}{\sqrt{\frac{r_2\sigma_C^2+(1-r_2)\sigma_T^2}{N(1-r_1)r_2(1-r_2)}\left(\mathrm{VAR}\left[Z_T\right]+\mathrm{VAR}\left[Z_C\right]+2\mathrm{COV}\left[Z_T,Z_C\right]\right)}} \\
=& \frac{1}{\sqrt{\frac{r_2\sigma_C^2+(1-r_2)\sigma_T^2}{N(1-r_1)r_2(1-r_2)}\left(\mathrm{VAR}\left[Z_T\right]+\mathrm{VAR}\left[Z_C\right]+2\mathrm{COV}\left[Z_T,Z_C\right]\right)}} \\
&\Bigg(\underbrace{\mathrm{COV}\left[\frac{1}{n_T}\sum_{i=1}^{n_T} y_{i,T}, \frac{1}{n_{T_\oplus}}\sum_{i=1}^{n_{T_\oplus}} y_{i,T_\oplus}\right]}_{=0} - \mathrm{COV}\left[\frac{1}{n_T}\sum_{i=1}^{n_T} y_{i,T}, \frac{n_{T_\oplus}}{n_T}\sum_{i=1}^{n_T} y_{i,T}\right] \\
&+ \underbrace{\mathrm{COV}\left[\frac{1}{n_T}\sum_{i=1}^{n_T} y_{i,T}, \frac{1}{n_{C_\ominus}}\sum_{i=1}^{n_{C_\ominus}} y_{i,C_\ominus}\right]}_{=0} - \underbrace{\mathrm{COV}\left[\frac{1}{n_T}\sum_{i=1}^{n_T} y_{i,T}, \frac{n_{C_\ominus}}{n_C}\sum_{i=1}^{n_C} y_{i,C}\right]}_{=0} \\
&- \underbrace{\mathrm{COV}\left[\frac{1}{n_C}\sum_{i=1}^{n_C} y_{i,C}, \frac{1}{n_{T_\oplus}}\sum_{i=1}^{n_{T_\oplus}} y_{i,T_\oplus}\right]}_{=0} + \underbrace{\mathrm{COV}\left[\frac{1}{n_C}\sum_{i=1}^{n_C} y_{i,C}, \frac{n_{T_\oplus}}{n_T}\sum_{i=1}^{n_T} y_{i,T}\right]}_{=0} \\
&- \underbrace{\mathrm{COV}\left[\frac{1}{n_C}\sum_{i=1}^{n_C} y_{i,C}, \frac{1}{n_{C_\ominus}}\sum_{i=1}^{n_{C_\ominus}} y_{i,C_\ominus}\right]}_{=0} + \mathrm{COV}\left[\frac{1}{n_C}\sum_{i=1}^{n_C} y_{i,C}, \frac{n_{C_\ominus}}{n_C}\sum_{i=1}^{n_C} y_{i,C}\right]\Bigg)
\end{aligned}
$$

$$
\begin{aligned}
&= \frac{-\mathrm{COV}\left[\frac{1}{n_T}\sum_{i=1}^{n_T} y_{i,T}, \frac{n_{T\oplus}}{n_T}\sum_{i=1}^{n_T} y_{i,T}\right] + \mathrm{COV}\left[\frac{1}{n_C}\sum_{i=1}^{n_C} y_{i,C}, \frac{n_{C\ominus}}{n_C}\sum_{i=1}^{n_C} y_{i,C}\right]}{\sqrt{\frac{r_2\sigma_C^2+(1-r_2)\sigma_T^2}{N(1-r_1)r_2(1-r_2)}\left(\mathrm{VAR}\left[Z_T\right]+\mathrm{VAR}\left[Z_C\right]+2\mathrm{COV}\left[Z_T,Z_C\right]\right)}} \\
&= \frac{-\left(\mathrm{E}\left[n_{T\oplus}\left(\frac{1}{n_T}\sum_{i=1}^{n_T} y_{i,T}\right)^2\right]-\mathrm{E}\left[\frac{1}{n_T}\sum_{i=1}^{n_T} y_{i,T}\right]\mathrm{E}\left[n_{T\oplus}\frac{1}{n_T}\sum_{i=1}^{n_T} y_{i,T}\right]\right)}{\sqrt{\frac{r_2\sigma_C^2+(1-r_2)\sigma_T^2}{N(1-r_1)r_2(1-r_2)}\left(\mathrm{VAR}\left[Z_T\right]+\mathrm{VAR}\left[Z_C\right]+2\mathrm{COV}\left[Z_T,Z_C\right]\right)}} \\
&+ \frac{\left(\mathrm{E}\left[n_{C\ominus}\left(\frac{1}{n_C}\sum_{i=1}^{n_C} y_{i,C}\right)^2\right]-\mathrm{E}\left[\frac{1}{n_C}\sum_{i=1}^{n_C} y_{i,C}\right]\mathrm{E}\left[n_{C\ominus}\frac{1}{n_C}\sum_{i=1}^{n_C} y_{i,C}\right]\right)}{\sqrt{\frac{r_2\sigma_C^2+(1-r_2)\sigma_T^2}{N(1-r_1)r_2(1-r_2)}\left(\mathrm{VAR}\left[Z_T\right]+\mathrm{VAR}\left[Z_C\right]+2\mathrm{COV}\left[Z_T,Z_C\right]\right)}} \\
&= \frac{-\left(\mathrm{E}\left[n_{T\oplus}\right]\mathrm{E}\left[\left(\frac{1}{n_T}\sum_{i=1}^{n_T} y_{i,T}\right)^2\right]-\mathrm{E}\left[n_{T\oplus}\right]\mathrm{E}\left[\frac{1}{n_T}\sum_{i=1}^{n_T} y_{i,T}\right]^2\right)}{\sqrt{\frac{r_2\sigma_C^2+(1-r_2)\sigma_T^2}{N(1-r_1)r_2(1-r_2)}\left(\mathrm{VAR}\left[Z_T\right]+\mathrm{VAR}\left[Z_C\right]+2\mathrm{COV}\left[Z_T,Z_C\right]\right)}} \\
&+ \frac{\left(\mathrm{E}\left[n_{C\ominus}\right]\mathrm{E}\left[\left(\frac{1}{n_C}\sum_{i=1}^{n_C} y_{i,C}\right)^2\right]-\mathrm{E}\left[n_{C\ominus}\right]\mathrm{E}\left[\frac{1}{n_C}\sum_{i=1}^{n_C} y_{i,C}\right]^2\right)}{\sqrt{\frac{r_2\sigma_C^2+(1-r_2)\sigma_T^2}{N(1-r_1)r_2(1-r_2)}\left(\mathrm{VAR}\left[Z_T\right]+\mathrm{VAR}\left[Z_C\right]+2\mathrm{COV}\left[Z_T,Z_C\right]\right)}} \\
&= \frac{-\left(\mathrm{E}\left[n_{T\oplus}\right]\left(\mathrm{V}\left[\frac{1}{n_T}\sum_{i=1}^{n_T} y_{i,T}\right]+\mathrm{E}\left[\frac{1}{n_T}\sum_{i=1}^{n_T} y_{i,T}\right]^2\right)-\mathrm{E}\left[n_{T\oplus}\right]\mathrm{E}\left[\frac{1}{n_T}\sum_{i=1}^{n_T} y_{i,T}\right]^2\right)}{\sqrt{\frac{r_2\sigma_C^2+(1-r_2)\sigma_T^2}{N(1-r_1)r_2(1-r_2)}\left(\mathrm{VAR}\left[Z_T\right]+\mathrm{VAR}\left[Z_C\right]+2\mathrm{COV}\left[Z_T,Z_C\right]\right)}} \\
&+ \frac{\left(\mathrm{E}\left[n_{C\ominus}\right]\left(\mathrm{V}\left[\frac{1}{n_C}\sum_{i=1}^{n_C} y_{i,C}\right]+\mathrm{E}\left[\frac{1}{n_C}\sum_{i=1}^{n_C} y_{i,C}\right]\right)-\mathrm{E}\left[n_{C\ominus}\right]\mathrm{E}\left[\frac{1}{n_C}\sum_{i=1}^{n_C} y_{i,C}\right]^2\right)}{\sqrt{\frac{r_2\sigma_C^2+(1-r_2)\sigma_T^2}{N(1-r_1)r_2(1-r_2)}\left(\mathrm{VAR}\left[Z_T\right]+\mathrm{VAR}\left[Z_C\right]+2\mathrm{COV}\left[Z_T,Z_C\right]\right)}} \\
&= \frac{-\overbrace{\mathrm{E}\left[n_{T\oplus}\right]}^{(2)}\overbrace{\mathrm{V}\left[\frac{1}{n_T}\sum_{i=1}^{n_T} y_{i,T}\right]}^{(14)}+\overbrace{\mathrm{E}\left[n_{C\ominus}\right]}^{(4)}\overbrace{\mathrm{V}\left[\frac{1}{n_C}\sum_{i=1}^{n_C} y_{i,C}\right]}^{(16)}}{\sqrt{\frac{r_2\sigma_C^2+(1-r_2)\sigma_T^2}{N(1-r_1)r_2(1-r_2)}\left(\underbrace{\mathrm{VAR}\left[Z_T\right]}_{(40)}+\underbrace{\mathrm{VAR}\left[Z_C\right]}_{(41)}+2\underbrace{\mathrm{COV}\left[Z_T,Z_C\right]}_{(42)}\right)}}
\end{aligned}
\tag{44}
$$

$$
\begin{aligned}
\mathrm{COV}_{B,I} =& \mathrm{COV}\left[T_B,T_I\right] = \mathrm{COV}\left[\frac{Z_T-Z_C}{\sqrt{\mathrm{VAR}\left[Z_T-Z_C\right]}}, \frac{Z_T+Z_C}{\sqrt{\mathrm{VAR}\left[Z_T+Z_C\right]}}\right] = \frac{\mathrm{COV}\left[Z_T-Z_C, Z_T+Z_C\right]}{\sqrt{\mathrm{VAR}\left[Z_T-Z_C\right]}\sqrt{\mathrm{VAR}\left[Z_T+Z_C\right]}} \\
=& \frac{\mathrm{VAR}\left[Z_T\right]-\mathrm{VAR}\left[Z_C\right]}{\sqrt{\mathrm{VAR}\left[Z_T-Z_C\right]}\sqrt{\mathrm{VAR}\left[Z_T+Z_C\right]}}
\end{aligned}
\tag{45}
$$

4 Sample size calculation

4.1 Treatment effect

The required sample size for the treatment effect to achieve a power of $1-\beta$ using a two-sided significance level α can be calculated by solving the following equation for N:

$$\frac{\overbrace{\mathrm{E}\left[Z_{TR}-Z_{CR}\right]}^{(34)}}{\sqrt{\underbrace{\mathrm{VAR}\left[Z_{TR}-Z_{CR}\right]}_{(35)}}}-z_{1-\alpha/2}=z_{1-\beta} \tag{46}$$

4.2 Biomarker and interaction effect

The required sample size for the biomarker and the interaction effect to achieve a power of $1-\beta$ using a two-sided significance level α can be calculated by solving the following equations for N:

$$\frac{\mathrm{E}\left[Z_T-Z_C\right]}{\sqrt{\mathrm{VAR}\left[Z_T-Z_C\right]}}-z_{1-\alpha/2}=z_{1-\beta} \tag{47}$$

$$\frac{\mathrm{E}\left[Z_T+Z_C\right]}{\sqrt{\mathrm{VAR}\left[Z_T+Z_C\right]}}-z_{1-\alpha/2}=z_{1-\beta}. \tag{48}$$

As the only difference between the biomarker and the interaction effect is the sign of Z_C (being negative for the biomarker effect and positive for the interaction effect), we can simplify the derivations by writing:

$$\frac{\mathrm{E}\left[Z_T\mp Z_C\right]}{\sqrt{\mathrm{VAR}\left[Z_T\mp Z_C\right]}}-z_{1-\alpha/2}=z_{1-\beta} \tag{49}$$

Note that the expected value $\mathrm{E}\left[Z_T\mp Z_C\right]$ and the variance $\mathrm{VAR}\left[Z_T\mp Z_C\right]$ can be written as:

$$\mathrm{E}\left[Z_T\mp Z_C\right]=Nr_1\left(\theta_{T_\oplus}\mp\theta_{C_\ominus}-p_\oplus\mu_T\pm p_\ominus\mu_C\right) \tag{50}$$

$$\begin{aligned}\mathrm{VAR}\left[Z_T\mp Z_C\right]=&Nr_1^2\left(\frac{\sigma_{T_\oplus}^2+\sigma_{C_\ominus}^2+p_\oplus p_\ominus\left(\mu_T^2+\mu_C^2\right)-2\left(p_\ominus\mu_T\theta_{T_\oplus}p_\oplus\mu_C\theta_{C_\ominus}\right)}{r_1}+\frac{\left(1-r_2\right)p_\oplus^2\sigma_T^2+r_2p_\ominus^2\sigma_C^2}{\left(1-r_1\right)r_2\left(1-r_2\right)}\right)\\&+\frac{r_1r_2p_\oplus p_\ominus\sigma_C^2+r_1\left(1-r_2\right)p_\oplus p_\ominus\sigma_T^2}{\left(1-r_1\right)r_2\left(1-r_2\right)}\mp Nr_1\left(-\theta_{T_\oplus}\theta_{C_\ominus}-p_\oplus p_\ominus\mu_T\mu_C+p_\ominus\mu_C\theta_{T_\oplus}+p_\oplus\mu_T\theta_{C_\ominus}\right)\\=&Nr_1^2A+B\mp Nr_1C\end{aligned} \tag{51}$$

with

$$\begin{aligned}A=&\frac{\sigma_{T_\oplus}^2+\sigma_{C_\ominus}^2+p_\oplus p_\ominus\left(\mu_T^2+\mu_C^2\right)-2\left(p_\ominus\mu_T\theta_{T_\oplus}p_\oplus\mu_C\theta_{C_\ominus}\right)}{r_1}+\frac{\left(1-r_2\right)p_\oplus^2\sigma_T^2+r_2p_\ominus^2\sigma_C^2}{\left(1-r_1\right)r_2\left(1-r_2\right)}\\B=&\frac{r_1r_2p_\oplus p_\ominus\sigma_C^2+r_1\left(1-r_2\right)p_\oplus p_\ominus\sigma_T^2}{\left(1-r_1\right)r_2\left(1-r_2\right)}\\C=&-\theta_{T_\oplus}\theta_{C_\ominus}-p_\oplus p_\ominus\mu_T\mu_C+p_\ominus\mu_C\theta_{T_\oplus}+p_\oplus\mu_T\theta_{C_\ominus}\end{aligned}$$

Hence, we get

$$\begin{aligned}&\frac{Nr_1\left(\theta_{T_\oplus}\mp\theta_{C-\ominus}-p_\oplus\mu_T\pm p_\ominus\mu_C\right)}{\sqrt{Nr_1^2A+B\mp Nr_1C}}=z_{1-\alpha/2}+z_{1-\beta}\\\Leftrightarrow\quad&\frac{\left(Nr_1\right)^2}{Nr_1^2A+B\mp Nr_1C}=\left(\frac{z_{1-\alpha/2}+z_{1-\beta}}{\theta_{T_\oplus}\mp\theta_{C-\ominus}-p_\oplus\mu_T\pm p_\ominus\mu_C}\right)^2.\end{aligned} \tag{52}$$

Setting

$$D = \left(\frac{z_{1-\alpha/2} + z_{1-\beta}}{\theta_{T\oplus} \mp \theta_{C-\ominus} - p_{\oplus}\mu_T \pm p_{\ominus}\mu_C} \right)^2 \tag{53}$$

and dividing by r_1^2 ($r_1 \neq 0$), we get

$$N^2 - D\frac{r_1 A \mp C}{r_1} N - D\frac{B}{r_1^2} = 0$$

$$\Leftrightarrow \qquad N = D\frac{r_1 A \mp C}{2r_1} \pm \sqrt{D\left(\frac{r_1 A \mp C}{2r_1}\right) + D\frac{B}{r_1^2}} \tag{54}$$

5 Estimators for the variances of the test statistics

Let s_T^2 and s_C^2 be defined as

$$s_T^2 = \frac{1}{n_T - 1} \sum_{i=1}^{n_T} \left(y_{T,i} - \frac{1}{n_T} \sum_{k=1}^{n_T} y_{T,k} \right)^2 \tag{55}$$

$$s_C^2 = \frac{1}{n_C - 1} \sum_{i=1}^{n_C} \left(y_{C,i} - \frac{1}{n_C} \sum_{k=1}^{n_C} y_{C,k} \right)^2 \tag{56}$$

with

$$\mathrm{E}\left[s_T^2\right] = \sigma_T^2 \tag{57}$$

$$\mathrm{E}\left[s_C^2\right] = \sigma_C^2 \tag{58}$$

The variances and covariances of Z_T and Z_C can be estimated by:

$$\widehat{\mathrm{VAR}}\left[Z_T\right] = \frac{1}{Nr_1 - 1} \left(Nr_1 \sum_{i=1}^{n_{T\oplus}} y_{T\oplus,i}^2 - \left(\sum_{i=1}^{n_{T\oplus}} y_{T\oplus,i} \right)^2 + Nr_1 n_{T\oplus} \left(n_{T\oplus} - 1\right) \frac{s_T^2}{n_T} \right.$$
$$\left. + Nr_1 n_{T\oplus} \left(1 - \frac{n_{T\oplus}}{Nr_1}\right) \left(\frac{1}{n_T} \sum_{i=1}^{n_T} y_{T,i} \right)^2 - 2Nr_1 \left(1 - \frac{n_{T\oplus}}{Nr_1}\right) \left(\sum_{i=1}^{n_{T\oplus}} y_{T\oplus,i} \right) \left(\frac{1}{n_T} \sum_{i=1}^{n_T} y_{T,i} \right) \right) \tag{59}$$

$$\widehat{\mathrm{VAR}}\left[Z_C\right] = \frac{1}{Nr_1 - 1} \left(Nr_1 \sum_{i=1}^{n_{C\ominus}} y_{C\ominus,i}^2 - \left(\sum_{i=1}^{n_{C\ominus}} y_{C\ominus,i} \right)^2 + Nr_1 n_{C\ominus} \left(n_{C\ominus} - 1\right) \frac{s_C^2}{n_C} \right.$$
$$\left. + Nr_1 n_{C\ominus} \left(1 - \frac{n_{C\ominus}}{Nr_1}\right) \left(\frac{1}{n_C} \sum_{i=1}^{n_C} y_{C,i} \right)^2 - 2Nr_1 \left(1 - \frac{n_{C\ominus}}{Nr_1}\right) \left(\sum_{i=1}^{n_{C\ominus}} y_{C\ominus,i} \right) \left(\frac{1}{n_C} \sum_{i=1}^{n_C} y_{C,i} \right) \right) \tag{60}$$

$$\widehat{\mathrm{COV}}\left[Z_T, Z_C\right] = \frac{1}{Nr_1 - 1} \left(- \left(\sum_{i=1}^{n_{T\oplus}} y_{T\oplus,i} \right) \left(\sum_{i=1}^{n_{C\ominus}} y_{C\ominus,i} \right) + \left(\sum_{i=1}^{n_{T\oplus}} y_{T\oplus,i} \right) n_{C\ominus} \left(\frac{1}{n_C} \sum_{i=1}^{n_C} y_{C,i} \right) \right.$$
$$\left. + \left(\sum_{i=1}^{n_{C\ominus}} y_{C\ominus,i} \right) n_{T\oplus} \left(\frac{1}{n_T} \sum_{i=1}^{n_T} y_{T,i} \right) - n_{T\oplus} n_{C\ominus} \left(\frac{1}{n_T} \sum_{i=1}^{n_T} y_{T,i} \right) \left(\frac{1}{n_C} \sum_{i=1}^{n_C} y_{C,i} \right) \right) \tag{61}$$

In the following, we show that the above estimators are unbiased estimators for the respective quantities.

$$
\begin{aligned}
\mathrm{E}\left[\widehat{\mathrm{VAR}}\left[Z_T\right]\right] =& \mathrm{E}\left[\frac{1}{Nr_1-1}\left(Nr_1\sum_{i=1}^{n_{T\oplus}} y_{T\oplus,i}^2-\left(\sum_{i=1}^{n_{T\oplus}} y_{T\oplus,i}\right)^2+Nr_1 n_{T\oplus}\left(n_{T\oplus}-1\right)\frac{s_T^2}{n_T}\right.\right.\\
&\left.\left.+Nr_1 n_{T\oplus}\left(1-\frac{n_{T\oplus}}{Nr_1}\right)\left(\frac{1}{n_T}\sum_{i=1}^{n_T} y_{T,i}\right)^2-2Nr_1\left(1-\frac{n_{T\oplus}}{Nr_1}\right)\left(\sum_{i=1}^{n_{T\oplus}} y_{T\oplus,i}\right)\left(\frac{1}{n_T}\sum_{i=1}^{n_T} y_{T,i}\right)\right)\right]\\
=&\frac{1}{Nr_1-1}\left(Nr_1\mathrm{E}\left[\sum_{i=1}^{n_{T\oplus}} y_{T\oplus,i}^2\right]-\mathrm{E}\left[\left(\sum_{i=1}^{n_{T\oplus}} y_{T\oplus,i}\right)^2\right]+Nr_1\mathrm{E}\left[n_{T\oplus}\left(n_{T\oplus}-1\right)\frac{s_T^2}{n_T}\right]\right.\\
&\left.+Nr_1\mathrm{E}\left[n_{T\oplus}\left(1-\frac{n_{T\oplus}}{Nr_1}\right)\left(\frac{1}{n_T}\sum_{i=1}^{n_T} y_{T,i}\right)^2\right]-2Nr_1\mathrm{E}\left[\left(1-\frac{n_{T\oplus}}{Nr_1}\right)\left(\sum_{i=1}^{n_{T\oplus}} y_{T\oplus,i}\right)\left(\frac{1}{n_T}\sum_{i=1}^{n_T} y_{T,i}\right)\right]\right)\\
=&\frac{1}{Nr_1-1}\left(Nr_1\mathrm{E}\left[\sum_{i=1}^{n_{T\oplus}} y_{T\oplus,i}^2\right]-\mathrm{E}\left[\sum_{i=1}^{n_{T\oplus}} y_{T\oplus,i}\right]^2-\mathrm{VAR}\left[\sum_{i=1}^{n_{T\oplus}} y_{T\oplus,i}\right]+Nr_1\mathrm{E}\left[n_{T\oplus}^2-n_{T\oplus}\right]\frac{1}{n_T}\mathrm{E}\left[s_T^2\right]\right.\\
&+Nr_1\mathrm{E}\left[n_{T\oplus}-\frac{1}{Nr_1}n_{T\oplus}^2\right]\mathrm{E}\left[\left(\frac{1}{n_T}\sum_{i=1}^{n_T} y_{T,i}\right)^2\right]\\
&\left.-2Nr_1\mathrm{E}\left[\left(1-\frac{n_{T\oplus}}{Nr_1}\right)\left(\sum_{i=1}^{n_{T\oplus}} y_{T\oplus,i}\right)\right]\mathrm{E}\left[\frac{1}{n_T}\sum_{i=1}^{n_T} y_{T,i}\right]\right)\\
=&\frac{1}{Nr_1-1}\left(Nr_1\underbrace{\mathrm{E}\left[\sum_{i=1}^{n_{T\oplus}} y_{T\oplus,i}^2\right]}_{(9)}-\underbrace{\mathrm{E}\left[\sum_{i=1}^{n_{T\oplus}} y_{T\oplus,i}\right]^2}_{(7)}-\underbrace{\mathrm{VAR}\left[\sum_{i=1}^{n_{T\oplus}} y_{T\oplus,i}\right]}_{(8)}\right.\\
&+Nr_1\left(\underbrace{\mathrm{E}\left[n_{T\oplus}\right]^2}_{(2)}+\underbrace{\mathrm{VAR}\left[n_{T\oplus}\right]}_{(3)}-\underbrace{\mathrm{E}\left[n_{T\oplus}\right]}_{(2)}\right)\frac{1}{n_T}\underbrace{\mathrm{E}\left[s_T^2\right]}_{(57)}\\
&+Nr_1\left(\underbrace{\mathrm{E}\left[n_{T\oplus}\right]}_{(2)}-\frac{1}{Nr_1}\left(\underbrace{\mathrm{E}\left[n_{T\oplus}\right]^2}_{(2)}+\underbrace{\mathrm{VAR}\left[n_{T\oplus}\right]}_{(3)}\right)\right)\left(\underbrace{\mathrm{E}\left[\frac{1}{n_T}\sum_{i=1}^{n_T} y_{T,i}\right]^2}_{(13)}+\underbrace{\mathrm{VAR}\left[\frac{1}{n_T}\sum_{i=1}^{n_T} y_{T,i}\right]}_{(14)}\right)\\
&\left.-2Nr_1\left(\underbrace{\mathrm{E}\left[\sum_{i=1}^{n_{T\oplus}} y_{T\oplus,i}\right]}_{(7)}-\frac{1}{Nr_1}\underbrace{\mathrm{E}\left[n_{T\oplus}\sum_{i=1}^{n_{T\oplus}} y_{T\oplus,i}\right]}_{(21)}\right)\underbrace{\mathrm{E}\left[\frac{1}{n_T}\sum_{i=1}^{n_T} y_{T,i}\right]}_{(13)}\right) \qquad (62)
\end{aligned}
$$

$$
\begin{aligned}
\mathrm{E}\left[\widehat{\mathrm{VAR}}\left[Z_C\right]\right] =& \mathrm{E}\left[\frac{1}{Nr_1-1}\left(Nr_1\sum_{i=1}^{n_{C_\ominus}} y_{C_\ominus,i}^2 - \left(\sum_{i=1}^{n_{C_\ominus}} y_{C_\ominus,i}\right)^2 + Nr_1 n_{C_\ominus}\left(n_{C_\ominus}-1\right)\frac{s_C^2}{n_C}\right.\right.\\
&\left.\left. + Nr_1 n_{C_\ominus}\left(1-\frac{n_{C_\ominus}}{Nr_1}\right)\left(\frac{1}{n_C}\sum_{i=1}^{n_C} y_{C,i}\right)^2 - 2Nr_1\left(1-\frac{n_{C_\ominus}}{Nr_1}\right)\left(\sum_{i=1}^{n_{C_\ominus}} y_{C_\ominus,i}\right)\left(\frac{1}{n_C}\sum_{i=1}^{n_C} y_{C,i}\right)\right)\right]\\
=& \frac{1}{Nr_1-1}\left(Nr_1\mathrm{E}\left[\sum_{i=1}^{n_{C_\ominus}} y_{C_\ominus,i}^2\right] - \mathrm{E}\left[\left(\sum_{i=1}^{n_{C_\ominus}} y_{C_\ominus,i}\right)^2\right] + Nr_1\mathrm{E}\left[n_{C_\ominus}\left(n_{C_\ominus}-1\right)\frac{s_C^2}{n_C}\right]\right.\\
&\left. + Nr_1\mathrm{E}\left[n_{C_\ominus}\left(1-\frac{n_{C_\ominus}}{Nr_1}\right)\left(\frac{1}{n_C}\sum_{i=1}^{n_C} y_{C,i}\right)^2\right] - 2Nr_1\mathrm{E}\left[\left(1-\frac{n_{C_\ominus}}{Nr_1}\right)\left(\sum_{i=1}^{n_{C_\ominus}} y_{C_\ominus,i}\right)\left(\frac{1}{n_C}\sum_{i=1}^{n_C} y_{C,i}\right)\right]\right)\\
=& \frac{1}{Nr_1-1}\left(Nr_1\mathrm{E}\left[\sum_{i=1}^{n_{C_\ominus}} y_{C_\ominus,i}^2\right] - \mathrm{E}\left[\sum_{i=1}^{n_{C_\ominus}} y_{C_\ominus,i}\right]^2 - \mathrm{VAR}\left[\sum_{i=1}^{n_{C_\ominus}} y_{C_\ominus,i}\right] + Nr_1\mathrm{E}\left[n_{C_\ominus}^2 - n_{C_\ominus}\right]\frac{1}{n_C}\mathrm{E}\left[s_C^2\right]\right.\\
& + Nr_1\mathrm{E}\left[n_{C_\ominus} - \frac{1}{Nr_1}n_{C_\ominus}^2\right]\mathrm{E}\left[\left(\frac{1}{n_C}\sum_{i=1}^{n_C} y_{C,i}\right)^2\right]\\
&\left. -2Nr_1\mathrm{E}\left[\left(1-\frac{n_{C_\ominus}}{Nr_1}\right)\left(\sum_{i=1}^{n_{C_\ominus}} y_{C_\ominus,i}\right)\right]\mathrm{E}\left[\frac{1}{n_C}\sum_{i=1}^{n_C} y_{C,i}\right]\right)\\
=& \frac{1}{Nr_1-1}\left(Nr_1\underbrace{\mathrm{E}\left[\sum_{i=1}^{n_{C_\ominus}} y_{C_\ominus,i}^2\right]}_{(12)} - \underbrace{\mathrm{E}\left[\sum_{i=1}^{n_{C_\ominus}} y_{C_\ominus,i}\right]^2}_{(10)} - \underbrace{\mathrm{VAR}\left[\sum_{i=1}^{n_{C_\ominus}} y_{C_\ominus,i}\right]}_{(11)}\right.\\
& + Nr_1\left(\underbrace{\mathrm{E}\left[n_{C_\ominus}\right]^2}_{(4)} + \underbrace{\mathrm{VAR}\left[n_{C_\ominus}\right]}_{(5)} - \underbrace{\mathrm{E}\left[n_{C_\ominus}\right]}_{(4)}\right)\frac{1}{n_C}\underbrace{\mathrm{E}\left[s_C^2\right]}_{(58)}\\
& + Nr_1\left(\underbrace{\mathrm{E}\left[n_{C_\ominus}\right]}_{(4)} - \frac{1}{Nr_1}\left(\underbrace{\mathrm{E}\left[n_{C_\ominus}\right]^2}_{(4)} + \underbrace{\mathrm{VAR}\left[n_{C_\ominus}\right]}_{(5)}\right)\right)\left(\underbrace{\mathrm{E}\left[\frac{1}{n_C}\sum_{i=1}^{n_C} y_{C,i}\right]^2}_{(15)} + \underbrace{\mathrm{VAR}\left[\frac{1}{n_C}\sum_{i=1}^{n_C} y_{C,i}\right]}_{(16)}\right)\\
&\left. -2Nr_1\left(\underbrace{\mathrm{E}\left[\sum_{i=1}^{n_{C_\ominus}} y_{C_\ominus,i}\right]}_{(10)} - \frac{1}{Nr_1}\underbrace{\mathrm{E}\left[n_{C_\ominus}\sum_{i=1}^{n_{C_\ominus}} y_{C_\ominus,i}\right]}_{(22)}\right)\underbrace{\mathrm{E}\left[\frac{1}{n_C}\sum_{i=1}^{n_C} y_{C,i}\right]}_{(15)}\right) \qquad (63)
\end{aligned}
$$

$$\begin{aligned}\mathrm{E}\left[\widehat{\mathrm{COV}}\left[Z_T, Z_C\right]\right] =&\mathrm{E}\left[\frac{1}{Nr_1-1}\left(-\left(\sum_{i=1}^{n_{T_\oplus}} y_{T_\oplus,i}\right)\left(\sum_{i=1}^{n_{C_\ominus}} y_{C_\ominus,i}\right)+\left(\sum_{i=1}^{n_{T_\oplus}} y_{T_\oplus,i}\right) n_{C_\ominus}\left(\frac{1}{n_C}\sum_{i=1}^{n_C} y_{C,i}\right)\right.\right.\\
&\left.\left.+\left(\sum_{i=1}^{n_{C_\ominus}} y_{C_\ominus,i}\right) n_{T_\oplus}\left(\frac{1}{n_T}\sum_{i=1}^{n_T} y_{T,i}\right)-n_{T_\oplus}n_{C_\ominus}\left(\frac{1}{n_T}\sum_{i=1}^{n_T} y_{T,i}\right)\left(\frac{1}{n_C}\sum_{i=1}^{n_C} y_{C,i}\right)\right)\right]\\
=&\frac{1}{Nr_1-1}\left(-\mathrm{E}\left[\left(\sum_{i=1}^{n_{T_\oplus}} y_{T_\oplus,i}\right)\left(\sum_{i=1}^{n_{C_\ominus}} y_{C_\ominus,i}\right)\right]+\mathrm{E}\left[\left(\sum_{i=1}^{n_{T_\oplus}} y_{T_\oplus,i}\right) n_{C_\ominus}\left(\frac{1}{n_C}\sum_{i=1}^{n_C} y_{C,i}\right)\right]\right.\\
&\left.+\mathrm{E}\left[\left(\sum_{i=1}^{n_{C_\ominus}} y_{C_\ominus,i}\right) n_{T_\oplus}\left(\frac{1}{n_T}\sum_{i=1}^{n_T} y_{T,i}\right)\right]-\mathrm{E}\left[n_{T_\oplus}n_{C_\ominus}\left(\frac{1}{n_T}\sum_{i=1}^{n_T} y_{T,i}\right)\left(\frac{1}{n_C}\sum_{i=1}^{n_C} y_{C,i}\right)\right]\right)\\
=&\frac{1}{Nr_1-1}\left(-\underbrace{\mathrm{E}\left[\left(\sum_{i=1}^{n_{T_\oplus}} y_{T_\oplus,i}\right)\left(\sum_{i=1}^{n_{C_\ominus}} y_{C_\ominus,i}\right)\right]}_{(25)}+\underbrace{\mathrm{E}\left[\left(\sum_{i=1}^{n_{T_\oplus}} y_{T_\oplus,i}\right) n_{C_\ominus}\right]}_{(23)}\underbrace{\mathrm{E}\left[\frac{1}{n_C}\sum_{i=1}^{n_C} y_{C,i}\right]}_{(15)}\right.\\
&\left.+\underbrace{\mathrm{E}\left[\left(\sum_{i=1}^{n_{C_\ominus}} y_{C_\ominus,i}\right) n_{T_\oplus}\right]}_{(24)}\underbrace{\mathrm{E}\left[\frac{1}{n_T}\sum_{i=1}^{n_T} y_{T,i})\right]}_{(13)}-\underbrace{\mathrm{E}\left[n_{T_\oplus}n_{C_\ominus}\right]}_{(6)}\underbrace{\mathrm{E}\left[\frac{1}{n_T}\sum_{i=1}^{n_T} y_{T,i}\right]}_{(13)}\underbrace{\mathrm{E}\left[\frac{1}{n_C}\sum_{i=1}^{n_C} y_{C,i}\right]}_{(15)}\right)
\end{aligned} \tag{64}$$

6 Confidence intervals

In the following, we assume that the true values for the prevalence p, the sensitivity t, and the specificity s are **known**. We want to estimate the differences $\mu_{T_+} - \mu_{C_+}$ and $\mu_{T_-} - \mu_{C_-}$. Note that the values of μ_{T_+}, μ_{C_+}, μ_{T_-}, and μ_{C_-} cannot be observed directly within this design. Let $\theta_{T_\oplus}$, $\theta_{C_\ominus}$, μ_T, and μ_C denote the true means of the biomarker-led treatment arm, the biomarker-led control arm, the randomised treatment arm, and the randomised control arm with

$$\theta_{T_\oplus} = pt\mu_{T_+} + (1-p)(1-s)\mu_{T_-}, \tag{65}$$
$$\theta_{C_\ominus} = p(1-t)\mu_{C_+} + (1-p)s\mu_{C_-}, \tag{66}$$
$$\mu_T = p\mu_{T_+} + (1-p)\mu_{T_-}, \tag{67}$$
$$\mu_C = p\mu_{C_+} + (1-p)\mu_{C_-}. \tag{68}$$

Solving the system of equations for μ_{T_+}, μ_{C_+}, μ_{T_-}, and μ_{C_-} yields

$$\mu_{T_+} = \frac{\theta_{T_\oplus} - (1-s)\mu_T}{p(t+s-1)}, \tag{69}$$
$$\mu_{C_+} = -\frac{\theta_{C_\ominus} - s\mu_C}{p(t+s-1)}, \tag{70}$$
$$\mu_{T_-} = -\frac{\theta_{T_\oplus} - t\mu_T}{(1-p)(t+s-1)}, \tag{71}$$
$$\mu_{C_-} = \frac{\theta_{C_\ominus} - (1-t)\mu_C}{(1-p)(t+s-1)}. \tag{72}$$

Hence, we can define point estimators as

$$\hat{\mu}_{T_+} - \hat{\mu}_{C_+} = \frac{\hat{\theta}_{T_\oplus} + \hat{\theta}_{C_\ominus} - (1-s)\hat{\mu}_T - s\hat{\mu}_C}{p(t+s-1)}, \tag{73}$$

$$\hat{\mu}_{T_-} - \hat{\mu}_{C_-} = -\frac{\hat{\theta}_{T_\oplus} + \hat{\theta}_{C_\ominus} - t\hat{\mu}_T - (1-t)\hat{\mu}_C}{(1-p)(t+s-1)} \tag{74}$$

with

$$\hat{\theta}_{T_\oplus} = \frac{1}{Nr_1}\sum_{i=1}^{n_{T_\oplus}} y_{T_\oplus,i}, \tag{75}$$

$$\hat{\theta}_{C_\ominus} = \frac{1}{Nr_1}\sum_{i=1}^{n_{C_\ominus}} y_{C_\ominus,i}, \tag{76}$$

$$\hat{\mu}_T = \frac{1}{n_T}\sum_{i=1}^{n_T} y_{T,i}, \tag{77}$$

$$\hat{\mu}_C = \frac{1}{n_C}\sum_{i=1}^{n_C} y_{C,i}. \tag{78}$$

The true variances and covariance of the estimators are given by

$$\begin{aligned}
\mathrm{VAR}\left[\hat{\mu}_{T_+} - \hat{\mu}_{C_+}\right] =& \mathrm{VAR}\left[\frac{\hat{\theta}_{T_\oplus} + \hat{\theta}_{C_\ominus} - (1-s)\hat{\mu}_T - s\hat{\mu}_C}{p(t+s-1)}\right] \\
=& \frac{1}{\left(p(t+s-1)\right)^2}\Big(\mathrm{VAR}\left[\hat{\theta}_{T_\oplus}\right] + \mathrm{VAR}\left[\hat{\theta}_{C_\ominus}\right] - (1-s)^2\mathrm{VAR}\left[\hat{\mu}_T\right] - s^2\mathrm{VAR}\left[\hat{\mu}_C\right] \\
&+ 2\mathrm{COV}\left[\hat{\theta}_{T_\oplus}, \hat{\theta}_{C_\ominus}\right] - 2(1-s)\underbrace{\mathrm{COV}\left[\hat{\theta}_{T_\oplus}, \hat{\mu}_T\right]}_{=0} - 2s\underbrace{\mathrm{COV}\left[\hat{\theta}_{T_\oplus}, \hat{\mu}_C\right]}_{=0} \\
&- 2(1-s)\underbrace{\mathrm{COV}\left[\hat{\theta}_{C_\ominus}, \hat{\mu}_T\right]}_{=0} + 2s\underbrace{\mathrm{COV}\left[\hat{\theta}_{C_\ominus}, \hat{\mu}_C\right]}_{=0} - 2s(1-s)\underbrace{\mathrm{COV}\left[\hat{\mu}_T, \hat{\mu}_C\right]}_{=0}\Big) \\
=& \frac{1}{\left(p(t+s-1)\right)^2}\Bigg(\frac{1}{(Nr_1)^2}\underbrace{\mathrm{VAR}\left[\sum_{i=1}^{n_{T_\oplus}} y_{T_\oplus,i}\right]}_{(8)} + \frac{1}{(Nr_1)^2}\underbrace{\mathrm{VAR}\left[\sum_{i=1}^{n_{C_\ominus}} y_{C_\ominus,i}\right]}_{(11)} \\
&- (1-s)^2\underbrace{\mathrm{VAR}\left[\frac{1}{n_T}\sum_{i=1}^{n_T} y_{T,i}\right]}_{(14)} - s^2\underbrace{\mathrm{VAR}\left[\frac{1}{n_C}\sum_{i=1}^{n_C} y_{C,i}\right]}_{(16)} + \frac{2}{(Nr_1)^2}\underbrace{\mathrm{COV}\left[\sum_{i=1}^{n_{T_\oplus}} y_{T_\oplus,i}, \sum_{i=1}^{n_{C_\ominus}} y_{C_\ominus,i}\right]}_{(28)}\Bigg) \\
=& \frac{1}{\left(p(t+s-1)\right)^2}\left(\frac{\sigma^2_{T_\oplus}}{Nr_1} + \frac{\sigma^2_{C_\ominus}}{Nr_1} + \frac{(1-s)^2\sigma^2_T}{n_T} + \frac{s^2\sigma^2_C}{n_C} - \frac{2\theta_{T_\oplus}\theta_{C_\ominus}}{Nr_1}\right)
\end{aligned} \tag{79}$$

$$\begin{aligned}
\mathrm{VAR}\left[\hat{\mu}_{T_-}-\hat{\mu}_{C_-}\right] =& \mathrm{VAR}\left[-\frac{\hat{\theta}_{T_\oplus}+\hat{\theta}_{C_\ominus}-t\hat{\mu}_T-(1-t)\hat{\mu}_C}{(1-p)(t+s-1)}\right] = (-1)^2\mathrm{VAR}\left[\frac{\hat{\theta}_{T_\oplus}+\hat{\theta}_{C_\ominus}-t\hat{\mu}_T-(1-t)\hat{\mu}_C}{(1-p)(t+s-1)}\right] \\
=&\frac{1}{\left((1-p)(t+s-1)\right)^2}\Big(\mathrm{VAR}\left[\hat{\theta}_{T_\oplus}\right]+\mathrm{VAR}\left[\hat{\theta}_{C_\ominus}\right]-t^2\mathrm{VAR}\left[\hat{\mu}_T\right]-(1-t)^2\mathrm{VAR}\left[\hat{\mu}_C\right] \\
&+2\mathrm{COV}\left[\hat{\theta}_{T_\oplus},\hat{\theta}_{C_\ominus}\right]-2t\underbrace{\mathrm{COV}\left[\hat{\theta}_{T_\oplus},\hat{\mu}_T\right]}_{=0}-2(1-t)\underbrace{\mathrm{COV}\left[\hat{\theta}_{T_\oplus},\hat{\mu}_C\right]}_{=0}-2t\underbrace{\mathrm{COV}\left[\hat{\theta}_{C_\ominus},\hat{\mu}_T\right]}_{=0} \\
&+2(1-t)\underbrace{\mathrm{COV}\left[\hat{\theta}_{C_\ominus},\hat{\mu}_C\right]}_{=0}-2t(1-t)\underbrace{\mathrm{COV}\left[\hat{\mu}_T,\hat{\mu}_C\right]}_{=0}\Big) \\
=&\frac{1}{\left((1-p)(t+s-1)\right)^2}\Bigg(\frac{1}{(Nr_1)^2}\underbrace{\mathrm{VAR}\left[\sum_{i=1}^{n_{T_\oplus}}y_{T_\oplus,i}\right]}_{(8)}+\frac{1}{(Nr_1)^2}\underbrace{\mathrm{VAR}\left[\sum_{i=1}^{n_{C_\ominus}}y_{C_\ominus,i}\right]}_{(11)} \\
&-t^2\underbrace{\mathrm{VAR}\left[\frac{1}{n_T}\sum_{i=1}^{n_T}y_{T,i}\right]}_{(14)}-(1-t)^2\underbrace{\mathrm{VAR}\left[\frac{1}{n_C}\sum_{i=1}^{n_C}y_{C,i}\right]}_{(16)}+\frac{2}{(Nr_1)^2}\underbrace{\mathrm{COV}\left[\sum_{i=1}^{n_{T_\oplus}}y_{T_\oplus,i},\sum_{i=1}^{n_{C_\ominus}}y_{C_\ominus,i}\right]}_{(28)}\Bigg) \\
=&\frac{1}{\left((1-p)(t+s-1)\right)^2}\left(\frac{\sigma^2_{T_\oplus}}{Nr_1}+\frac{\sigma^2_{C_\ominus}}{Nr1}+\frac{t^2\sigma^2_T}{n_T}+\frac{(1-t)^2\sigma^2_C}{n_C}-\frac{2\theta_{T_\oplus}\theta_{C_\ominus}}{Nr_1}\right)
\end{aligned} \tag{80}$$

$$\begin{aligned}
\mathrm{COV}\left[\hat{\mu}_{T_+}-\hat{\mu}_{C_+},\hat{\mu}_{T_-}-\hat{\mu}_{C_-}\right] =& -\frac{1}{p(1-p)(t+s-1)^2}\Big(\mathrm{V}\left[\hat{\theta}_{T_\oplus}\right]+\mathrm{V}\left[\hat{\theta}_{C_\ominus}\right]+t(1-s)\mathrm{V}\left[\hat{\mu}_T\right]+(1-t)s\mathrm{V}\left[\hat{\mu}_C\right] \\
&+\mathrm{COV}\left[\hat{\theta}_{T_\oplus},\hat{\theta}_{C_\ominus}\right]-(t+1-s)\underbrace{\mathrm{COV}\left[\hat{\theta}_{T_\oplus},\hat{\mu}_T\right]}_{=0}-(1-t+s)\underbrace{\mathrm{COV}\left[\hat{\theta}_{T_\oplus},\hat{\mu}_C\right]}_{=0} \\
&-(t+1-s)\underbrace{\mathrm{COV}\left[\hat{\theta}_{C_\ominus},\hat{\mu}_T\right]}_{=0}-(1-t+s)\underbrace{\mathrm{COV}\left[[\hat{\theta}_{C_\ominus},\hat{\mu}_C\right]}_{=0} \\
&+((1-t)(1-s)+ts)\underbrace{\mathrm{COV}\left[\hat{\mu}_T,\hat{\mu}_C\right]}_{=0}\Big) \\
=&-\frac{1}{p(1-p)(t+s-1)^2}\left(\frac{\sigma^2_{T_\oplus}}{Nr_1}+\frac{\sigma^2_{C_\ominus}}{Nr_1}+\frac{t(1-s)\sigma^2_T}{n_T}+\frac{(1-t)s\sigma^2_C}{n_C}-\frac{2\theta_{T_\oplus}\theta_{C_\ominus}}{Nr_1}\right)
\end{aligned} \tag{81}$$

These variances and the covariance can be estimated as follows:

$$\begin{aligned}
\widehat{\mathrm{VAR}}\left[\hat{\mu}_{T_+} - \hat{\mu}_{C_+}\right] =& \frac{1}{\left(p(t+s-1)\right)^2}\left(\frac{Nr_1 \sum_{i=1}^{n_{T\oplus}} y_{T\oplus,i}^2 - \left(\sum_{i=1}^{n_{T\oplus}} y_{T\oplus,i}\right)^2}{(Nr_1)^2(Nr_1-1)}\right.\\
&+\frac{Nr_1 \sum_{i=1}^{n_{C\ominus}} y_{C\ominus,i}^2 - \left(\sum_{i=1}^{n_{C\ominus}} y_{C\ominus,i}\right)^2}{(Nr_1)^2(Nr_1-1)} + \frac{(1-s)^2 s_T^2}{n_T} + \frac{s^2 s_C^2}{n_C}\\
&\left.-\frac{2}{(Nr_1)^2(Nr_1-1)}\left(\sum_{i=1}^{n_{T\oplus}} y_{T\oplus,i}\right)\left(\sum_{i=1}^{n_{C\ominus}} y_{C\ominus,i}\right)\right) \qquad (82)
\end{aligned}$$

$$\begin{aligned}
\widehat{\mathrm{VAR}}\left[\hat{\mu}_{T_-} - \hat{\mu}_{C_-}\right] =& \frac{1}{\left((1-p)(t+s-1)\right)^2}\left(\frac{Nr_1 \sum_{i=1}^{n_{T\oplus}} y_{T\oplus,i}^2 - \left(\sum_{i=1}^{n_{T\oplus}} y_{T\oplus,i}\right)^2}{(Nr_1)^2(Nr_1-1)}\right.\\
&+\frac{Nr_1 \sum_{i=1}^{n_{C\ominus}} y_{C\ominus,i}^2 - \left(\sum_{i=1}^{n_{C\ominus}} y_{C\ominus,i}\right)^2}{(Nr_1)^2(Nr_1-1)} + \frac{t^2 s_T^2}{n_T} + \frac{(1-t)^2 s_C^2}{n_C}\\
&\left.-\frac{2}{(Nr_1)^2(Nr_1-1)}\left(\sum_{i=1}^{n_{T\oplus}} y_{T\oplus,i}\right)\left(\sum_{i=1}^{n_{C\ominus}} y_{C\ominus,i}\right)\right) \qquad (83)
\end{aligned}$$

$$\begin{aligned}
\widehat{\mathrm{COV}}\left[\hat{\mu}_{T_+} - \hat{\mu}_{C_+}, \hat{\mu}_{T_-} - \hat{\mu}_{C_-}\right] =& \frac{1}{p(1-p)\left(t+s-1\right)^2}\left(\frac{Nr_1 \sum_{i=1}^{n_{T\oplus}} y_{T\oplus,i}^2 - \left(\sum_{i=1}^{n_{T\oplus}} y_{T\oplus,i}\right)^2}{(Nr_1)^2(Nr_1-1)}\right.\\
&+\frac{Nr_1 \sum_{i=1}^{n_{C\ominus}} y_{C\ominus,i}^2 - \left(\sum_{i=1}^{n_{C\ominus}} y_{C\ominus,i}\right)^2}{(Nr_1)^2(Nr_1-1)} + \frac{t(1-s) s_T^2}{n_T} + \frac{(1-t) s s_C^2}{n_C}\\
&\left.-\frac{2}{(Nr_1)^2(Nr_1-1)}\left(\sum_{i=1}^{n_{T\oplus}} y_{T\oplus,i}\right)\left(\sum_{i=1}^{n_{C\ominus}} y_{C\ominus,i}\right)\right) \qquad (84)
\end{aligned}$$

For the definition of s_T^2 and s_C^2 see Equations (55) and (56). In the following, we show that the above estimators are unbiased estimators for the respective quantities.

$$\begin{aligned}
\mathrm{E}\left[\widehat{\mathrm{VAR}}\left[\hat{\mu}_{T_+}-\hat{\mu}_{C_+}\right]\right] =& \mathrm{E}\left[\frac{1}{\left(p(t+s-1)\right)^2}\left(\frac{Nr_1\sum_{i=1}^{n_{T_\oplus}} y_{T_\oplus,i}^2-\left(\sum_{i=1}^{n_{T_\oplus}} y_{T_\oplus,i}\right)^2}{(Nr_1)^2(Nr_1-1)}\right.\right.\\
&+\frac{Nr_1\sum_{i=1}^{n_{C_\ominus}} y_{C_\ominus,i}^2-\left(\sum_{i=1}^{n_{C_\ominus}} y_{C_\ominus,i}\right)^2}{(Nr_1)^2(Nr_1-1)}+\frac{(1-s)^2s_T^2}{n_T}+\frac{s^2s_C^2}{n_C}\\
&\left.\left.-\frac{2}{(Nr_1)^2(Nr_1-1)}\left(\sum_{i=1}^{n_{T_\oplus}} y_{T_\oplus,i}\right)\left(\sum_{i=1}^{n_{C_\ominus}} y_{C_\ominus,i}\right)\right)\right]\\
=&\frac{1}{\left(p(t+s-1)\right)^2}\left(\frac{1}{(Nr_1)^2(Nr_1-1)}\left(Nr_1\overbrace{\mathrm{E}\left[\sum_{i=1}^{n_{T_\oplus}} y_{T_\oplus,i}^2\right]}^{(9)}-\overbrace{\mathrm{E}\left[\sum_{i=1}^{n_{T_\oplus}} y_{T_\oplus,i}\right]}^{(7)}{}^2-\overbrace{\mathrm{VAR}\left[\sum_{i=1}^{n_{T_\oplus}} y_{T_\oplus,i}\right]}^{(8)}\right)\right.\\
&+\frac{1}{(Nr_1)^2(Nr_1-1)}\left(Nr_1\overbrace{\mathrm{E}\left[\sum_{i=1}^{n_{C_\ominus}} y_{C_\ominus,i}^2\right]}^{(12)}-\overbrace{\mathrm{E}\left[\sum_{i=1}^{n_{C_\ominus}} y_{C_\ominus,i}\right]}^{(10)}{}^2-\overbrace{\mathrm{VAR}\left[\sum_{i=1}^{n_{C_\ominus}} y_{C_\ominus,i}\right]}^{(11)}\right)\\
&\left.+\frac{(1-s)^2\overbrace{\mathrm{E}\left[s_T^2\right]}^{(57)}}{n_T}+\frac{s^2\overbrace{\mathrm{E}\left[s_C^2\right]}^{(58)}}{n_C}-\frac{2}{(Nr_1)^2(Nr_1-1)}\overbrace{\mathrm{E}\left[\left(\sum_{i=1}^{n_{T_\oplus}} y_{T_\oplus,i}\right)\left(\sum_{i=1}^{n_{C_\ominus}} y_{C_\ominus,i}\right)\right]}^{(25)}\right)
\end{aligned} \tag{85}$$

$$\begin{aligned}
\mathrm{E}\left[\widehat{\mathrm{VAR}}\left[\hat{\mu}_{T_-}-\hat{\mu}_{C_-}\right]\right] =& \mathrm{E}\left[\frac{1}{\left((1-p)(t+s-1)\right)^2}\left(\frac{Nr_1\sum_{i=1}^{n_{T_\oplus}} y_{T_\oplus,i}^2-\left(\sum_{i=1}^{n_{T_\oplus}} y_{T_\oplus,i}\right)^2}{(Nr_1)^2(Nr_1-1)}\right.\right.\\
&+\frac{Nr_1\sum_{i=1}^{n_{C_\ominus}} y_{C_\ominus,i}^2-\left(\sum_{i=1}^{n_{C_\ominus}} y_{C_\ominus,i}\right)^2}{(Nr_1)^2(Nr_1-1)}+\frac{t^2s_T^2}{n_T}+\frac{(1-t)^2s_C^2}{n_C}\\
&\left.\left.-\frac{2}{(Nr_1)^2(Nr_1-1)}\left(\sum_{i=1}^{n_{T_\oplus}} y_{T_\oplus,i}\right)\left(\sum_{i=1}^{n_{C_\ominus}} y_{C_\ominus,i}\right)\right)\right]\\
=&\frac{1}{\left((1-p)(t+s-1)\right)^2}\left(\frac{1}{(Nr_1)^2(Nr_1-1)}\left(Nr_1\overbrace{\mathrm{E}\left[\sum_{i=1}^{n_{T_\oplus}} y_{T_\oplus,i}^2\right]}^{(9)}-\overbrace{\mathrm{E}\left[\sum_{i=1}^{n_{T_\oplus}} y_{T_\oplus,i}\right]}^{(7)}{}^2\right.\right.\\
&\left.-\overbrace{\mathrm{VAR}\left[\sum_{i=1}^{n_{T_\oplus}} y_{T_\oplus,i}\right]}^{(8)}\right)+\frac{1}{(Nr_1)^2(Nr_1-1)}\left(Nr_1\overbrace{\mathrm{E}\left[\sum_{i=1}^{n_{C_\ominus}} y_{C_\ominus,i}^2\right]}^{(12)}-\overbrace{\mathrm{E}\left[\sum_{i=1}^{n_{C_\ominus}} y_{C_\ominus,i}\right]}^{(10)}\right.\\
&\left.-\overbrace{\mathrm{VAR}\left[\sum_{i=1}^{n_{C_\ominus}} y_{C_\ominus,i}\right]}^{(11)}\right)+\frac{t^2\overbrace{\mathrm{E}\left[s_T^2\right]}^{(57)}}{n_T}+\frac{(1-t)^2\overbrace{\mathrm{E}\left[s_C^2\right]}^{(58)}}{n_C}\\
&\left.-\frac{2}{(Nr_1)^2(Nr_1-1)}\overbrace{\mathrm{E}\left[\left(\sum_{i=1}^{n_{T_\oplus}} y_{T_\oplus,i}\right)\left(\sum_{i=1}^{n_{C_\ominus}} y_{C_\ominus,i}\right)\right]}^{(25)}\right)
\end{aligned} \tag{86}$$

$$
\begin{aligned}
\mathrm{E}\left[\widehat{\mathrm{COV}}\left[\hat{\mu}_{T_+}-\hat{\mu}_{C_+},\hat{\mu}_{T_-}-\hat{\mu}_{C_-}\right]\right]=&\mathrm{E}\left[\frac{1}{p(1-p)\,(t+s-1)^2}\left(\frac{Nr_1\sum_{i=1}^{n_{T_\oplus}}y_{T_\oplus,i}^2-\left(\sum_{i=1}^{n_{T_\oplus}}y_{T_\oplus,i}\right)^2}{(Nr_1)^2(Nr_1-1)}\right.\right.\\
&+\frac{Nr_1\sum_{i=1}^{n_{C_\ominus}}y_{C_\ominus,i}^2-\left(\sum_{i=1}^{n_{C_\ominus}}y_{C_\ominus,i}\right)^2}{(Nr_1)^2(Nr_1-1)}+\frac{t(1-s)s_T^2}{n_T}+\frac{(1-t)ss_C^2}{n_C}\\
&\left.\left.-\frac{2}{(Nr_1)^2(Nr_1-1)}\left(\sum_{i=1}^{n_{T_\oplus}}y_{T_\oplus,i}\right)\left(\sum_{i=1}^{n_{C_\ominus}}y_{C_\ominus,i}\right)\right)\right]\\
=&\frac{1}{p(1-p)\,(t+s-1)^2}\left(\frac{1}{(Nr_1)^2(Nr_1-1)}\left(Nr_1\overbrace{\mathrm{E}\left[\sum_{i=1}^{n_{T_\oplus}}y_{T_\oplus,i}^2\right]}^{(9)}-\overbrace{\mathrm{E}\left[\sum_{i=1}^{n_{T_\oplus}}y_{T_\oplus,i}\right]}^{(7)}{}^2\right.\right.\\
&\left.-\overbrace{\mathrm{VAR}\left[\sum_{i=1}^{n_{T_\oplus}}y_{T_\oplus,i}\right]}^{(8)}\right)+\frac{1}{(Nr_1)^2(Nr_1-1)}\left(Nr_1\overbrace{\mathrm{E}\left[\sum_{i=1}^{n_{C_\ominus}}y_{C_\ominus,i}^2\right]}^{(12)}-\overbrace{\mathrm{E}\left[\sum_{i=1}^{n_{C_\ominus}}y_{C_\ominus,i}\right]}^{(10)}\right.\\
&\left.-\overbrace{\mathrm{VAR}\left[\sum_{i=1}^{n_{C_\ominus}}y_{C_\ominus,i}\right]}^{(11)}\right)+\frac{t(1-s)\overbrace{\mathrm{E}\left[s_T^2\right]}^{(57)}}{n_T}+\frac{(1-t)s\overbrace{\mathrm{E}\left[s_C^2\right]}^{(58)}}{n_C}\\
&\left.-\frac{2}{(Nr_1)^2(Nr_1-1)}\overbrace{\mathrm{E}\left[\left(\sum_{i=1}^{n_{T_\oplus}}y_{T_\oplus,i}\right)\left(\sum_{i=1}^{n_{C_\ominus}}y_{C_\ominus,i}\right)\right]}^{(25)}\right) \qquad (87)
\end{aligned}
$$
